# Overexpression of soybean *DREB1* enhances drought stress tolerance of transgenic wheat in the field

**DOI:** 10.1093/jxb/erz569

**Published:** 2019-12-26

**Authors:** Yongbin Zhou, Ming Chen, Jinkao Guo, Yanxia Wang, Donghong Min, Qiyan Jiang, Hutai Ji, Chengyan Huang, Wei Wei, Huijun Xu, Xiao Chen, Liancheng Li, Zhaoshi Xu, Xianguo Cheng, Chunxiao Wang, Chengshe Wang, Youzhi Ma

**Affiliations:** 1 College of Agronomy, Northwest A&F University, Yangling, Shaanxi, China; 2 Institute of Crop Sciences, Chinese Academy of Agricultural Sciences (CAAS)/National Key Facility for Crop Gene Resources and Genetic Improvement, Key Laboratory of Biology and Genetic Improvement of Triticeae Crops, Ministry of Agriculture, Beijing, China; 3 Shijiazhuang Academy of Agricultural and Forestry Sciences, Research Center of Wheat Engineering Technology of Hebei, Shijiazhuang, Hebei, China; 4 Institute of Wheat Research, Shanxi Academy of Agricultural Sciences, Shanxi, China; 5 Crop Research Institute, Shangdong Academy of Agricultural Sciences, Shandong, China; 6 CSIRO Agriculture and Food, Australia

**Keywords:** DREB-like transcription factor, drought stress tolerance, grain yield, melatonin, physiological traits, transgenic wheat

## Abstract

Drought-response-element binding (DREB)-like transcription factors can significantly enhance plant tolerance to water stress. However, most research on DREB-like proteins to date has been conducted in growth chambers or greenhouses, so there is very little evidence available to support their practical use in the field. In this study, we overexpressed *GmDREB1* from soybean in two popular wheat varieties and conducted drought-tolerance experiments across a range of years, sites, and drought-stress regimes. We found that the transgenic plants consistently exhibited significant improvements in yield performance and a variety of physiological traits compared with wild-type plants when grown under limited water conditions in the field, for example showing grain yield increases between 4.79–18.43%. Specifically, we found that the transgenic plants had reduced membrane damage and enhanced osmotic adjustment and photosynthetic efficiency compared to the non-transgenic controls. Three enzymes from the biosynthetic pathway of the phytohormone melatonin were up-regulated in the transgenic plants, and external application of melatonin was found to improve drought tolerance. Together, our results demonstrate the utility of transgenic overexpression of *GmDREB1* to improve the drought tolerance of wheat in the field.

## Teaser

Overexpression of soybean *GmDREB1* in wheat confers drought tolerance in the field and is associated with increased melatonin content, an increased root system, and delayed leaf senescence.

## Introduction

Water deficit is one of the most significant abiotic stresses for crop growth, development, and productivity (Saad *et al.*, 2013; Thirumalaikumar *et al.*, 2018). The North China Plain (NCP) is one of the most productive agricultural areas of the country, with more than 50% of domestic wheat grain being produced in this region (Wang *et al.*, 2012). As a result of recent very serious rainfall shortages and the consequent over-exploitation of groundwater during the wheat-growing season, the groundwater level in the NCP has been seriously reduced (Zhang *et al.*, 1999; Wu *et al.*, 2006; Fang *et al.*, 2010, 2017; Xie *et al.*, 2017). Hence there is an urgent need to develop wheat varieties that exhibit drought tolerance or other water-saving traits in order to mitigate the effects of the ongoing water shortage.

To date, many important genes controlling drought resistance have been identified in plants. The drought-response-element binding (DREB) transcription factor family is found in multiple plant species, and has been shown to function in enhancing plant tolerance to various abiotic stresses, including drought stress (Liu *et al.*, 1998; Agarwal *et al.*, 2006). In Arabidopsis, DREB1/CBF (C-repeat binding factor) proteins are thought to function in the expression of cold-responsive genes, and the DREB2 protein is a known positive regulator of drought-responsive gene expression (Sakuma *et al.*, 2006). Overexpression of the soybean transcription factor *DREB1* improves tolerance to heat and drought stress, as well as cold stress, in transgenic Arabidopsis (Kidokoro *et al.*, 2015). While it is now accepted that the transgenic expression of *DREB*-like genes can improve the drought tolerance of a variety of crops, it is notable that most of the studies examining such effects have been conducted in greenhouses, rather than in field conditions that could present more relevant and useful information about the basic physiology and yield -related performance of the transgenic expression of these genes.

Melatonin (N-acetyl-5-methoxytrytamine) is a low-molecular-weight indoleamine mammalian neurotransmitter that has also been identified as an important multi-functional signaling molecule in plants (Erland *et al.*, 2018). Melatonin is involved in plant responses to environmental stresses, including drought, salt, and heavy metal stress (Li *et al.*, 2012; Bajwa *et al.*, 2014). However, to date most of the components in melatonin-related signaling pathways in plants remain largely unknown.

In a previous study, we characterized the *GmDREB1* gene from soybean (*Glycine max*) and found that its overexpression improved the drought resistance of transgenic wheat (*Triticum aestivum* cv. Lumai22) under greenhouse conditions (Gao *et al.*, 2005). Hypothesizing that *GmDREB1* may also confer drought-stress tolerance in other wheat varieties, in the current study we introduced it into two popular wheat varieties and found that its overexpression did indeed significantly improve performance under drought stress. We then moved to field conditions and found that the *GmDREB1* transgenic plants also exhibited improvements in grain yield and in a variety of known yield-related and physiological traits. Subsequent examination of photosynthetic capacity indicated that the transgenic plants had enhanced photosynthesis that apparently resulted from prolonged leaf functional duration. Gene expression profiling suggested that altered melatonin metabolism was implicated in the observed drought-related effects, and the influence of this hormone on the observed differences in growth under drought stress was confirmed via chemical profiling of melatonin content in transgenic and wild-type plants. In addition to this potential role for melatonin in regulating plant abiotic stress responses, our work provides a convincing demonstration of the utility of overexpression of *GmDREB1* to improve the drought tolerance of wheat in the field.

## Materials and methods

### Wheat transformation

*GmDREB1* (GenBank no. AF514908; Glyma.14G084700) from *Glycine max* was inserted into the plasmid pAHC25 under the control of the ubiquitin (Ubi) promoter. Transgenic wheat (*Triticum aestivum*) plants of the varieties Jimai19 and Jimai20 were generated using the particle bombardment method (Xu *et al.*, 2007), and *bar*, a herbicide-resistance gene, was used as the plant selection marker gene. All T_0_ regenerated plantlets were potted into soil and grown in a greenhouse. A total of 170 regenerated plantlets were obtained from 1715 Jimai19 calli, and 14 regenerated plantlets were obtained from 490 Jimai20 calli. After PCR-based genotyping, 51 positive transgenic lines were identified in the Jimai19 genetic background and four in the Jimai20 background, so the transformation efficiencies were 3.0% and 0.8%, respectively (Supplementary Table S1 at *JXB* online). The T_0_ to T_3_ generations of the transgenic plants were tracked using PCR-based genotyping and semi-quantitative RT-PCR. We extracted genomic DNA using the CTAB method (Murray and Thompson, 1980). The specific primers for *GmDREB1* are listed in Supplementary Table S15 (*UBI-*F from the vector, *GmDREB1*-R). The *GmDREB1* copy number was examined using a Southern blotting protocol (Southern, 1975) in each of the transgenic lines that we examined in the field in this study (T349, T398, CM7, and CM14; see Supplementary Table S2 for details of the lines). The transcript levels of *GmDREB1* in the T_4_ generation of the transgenic lines were measured using semi-quantitative PCR (Shafi *et al.*, 2015), with the primers listed in Supplementary Table S15.

### Analysis of drought tolerance in the greenhouse

In order to characterize the drought tolerance of the transgenic wheat lines in the greenhouse (Supplementary Fig. S1), seeds of the T_2_ generation were selected and sown in boxes (65×35×15 cm) under conditions of 16/8 h light/dark at 20 °C, 300 μmol m^–2^ s^–1^. Each experiment was repeated three times. When the germinated seedlings had grown for 3 weeks, we stopped watering for 45 d, followed by rehydration for 20 d, and the survival rates of the plants were determined (Supplementary Fig. S2). In the T_4_ generation, three homozygous transgenic lines, namely T349, T378, and T398, and the wild-type (WT, Jimai19) were sown in boxes (55×35×10 cm, 50 seeds for each line) under a 16/8 h light/dark cycle at 20 °C, 300 μmol m^–2^ s^–1^. When the third leaf was visible, we stopped watering for 21 d until the soil moisture reached 40–45%, and then rehydrated for 7 d to a relative soil water content of 75–80%. These experiments included a control group for which the soil moisture was maintained at 75–80% for 28 d. We observed the degree of leaf curling after cessation of watering for 7 d, and also determined the survival rates of all the lines. The water loss rates of leaves from the plants were determined in relation to untreated control leaves, as previously described by Yu *et al.* (2017).

### Analysis of drought tolerance in the field

We conducted three years of field testing in three major wheat-growing areas in the North China Plain (NCP): Shijiazhuang in Hebei province (37°24′11″N, 114°25′53″E); Jinan in Shandong province (36°42′13″N, 117°04′23″E); and Linfen in Shanxi province (36°14′48″N, 111°58′09″E). It should be noted that rainfall in these three NCP regions was very limited; a survey of precipitation in Shijiazhuang, Jinan, and Linfen from 2010 to 2013 (Supplementary Fig. S11) showed that the soil moisture content in the 0–40 cm zone in the field remained at 10–18%, which was equivalent to moderate drought stress (Supplementary Table S12). For these field experiments, *GmDREB1* transgenic and WT plants were sown on 2 October each year (2010–2012) and harvested in the following June (2011–2013). The experimental plots were 6.7 m^2^, with three replicates in each area. Three water treatments were applied in the experiments following previous field research drought stress protocols (Reza *et al.*, 2009): briefly, 50 m^3^ of water were applied for each of the following irrigation events. In the limited-irrigation (LIR) treatment, an additional irrigation was applied during the jointing stage at the Shijiazhuang and Jinan sties, and an additional irrigation was applied during each of the pre-wintering and jolting stages at the Linfen site. In the well-irrigated (WIR) treatment, an additional irrigation was applied during each of the jointing and the filling stages at Shijiazhuang and Jinan, and at the Linfen site a third irrigation was also applied during the pre-wintering stage. In the non-irrigated (NIR) treatment, no additional irrigation was applied from sowing to harvest.: Other crop management was consistent with local cultivation practices for wheat varieties in the field. The major agronomic traits of the transgenic wheat and the WT were investigated based on plants randomly selected from the plots. All of the wheat lines were evaluated using the stress susceptibility index (SSI; Fischer and Maurer, 1978):

$$\begin{array}{l} SSI=\left[1\left(Y_{s}/Y_{p}\right)\right]/\left[1\left({\bar{Y}}_{s}/{\bar{Y}}_{p}\right)\right] \end{array}$$

where *Y*_s_ and *Y*_p_ are the indices of transgenic and WT lines evaluated under stress and normal conditions, respectively, and ${\bar{Y}}_{s}$ and ${\bar{Y}}_{p}$ are the mean yields over all the transgenic and WT lines evaluated under stress and non-stress conditions, respectively.

### Water use efficiency

Grain water use efficiency (WUE; kg ha^–1^ mm^–1^) was defined as according to Liu *et al.* (2018):

$$\begin{array}{l} WUE=GY/ET \end{array}$$

where GY is grain yield (kg ha– ^1^) and ET is evapotranspiration (mm). The latter was calculated using the field water balance equation (Yang *et al.*, 2011):

$$\begin{array}{l} ET=I+P+U-D-R-\Delta S \end{array}$$

where *I* is the amount of irrigation water applied (mm), *P* is the amount of precipitation (mm), *R* is surface run-off (mm; because of strict control of irrigation during the growing seasons, run-off was never observed in the field, so this variable was ignored), And *U* is the upward capillary flow into the root zone (mm; this was regarded as negligible, according to Liu *et al.*, 2018). *D* is the downward drainage from the root zone (mm). Previous studies have indicated that drainage from the root zone can be ignored in the North China Plain, including at our experimental site (Lv *et al.*, 2011; Xie *et al.*, 2017). ΔS is the change in soil water storage over a given time interval (mm) and was calculated as previously described by Liu *et al.* (2018).

### Measurements of drought-related physiological characteristics in the field, and under rainproof shelters

After the flowering stage in the field, 10 individual plants of the transgenic line T349 were randomly sampled for each of the three irrigation treatments (WIR, LIR, and NIR). Four traits were evaluated, namely the contents of malondialdehyde (MDA), proline, total soluble proteins, and chlorophyll, and plants were sampled from each of the three replicate plots in each experiment.

For the rainproof-shelter experiments, after emergence seedlings were transferred into plastic pots (25×45×35 cm) filled with surface soil. All the pot experiments were divided into two groups, as follows. In the drought treatment group, the plants of the T349 and T398 transgenic lines and the WT (Jimai19 genetic background) were subjected to drought treatment (relative soil water content of 40–45%) at either the jointing stage, heading stage, flowering stage, or grain-filling stage. In the normal treatment group, plants of the T349 and T398 transgenic lines and the WT (Jimai19 background) were provided with an adequate water supply (relative soil water content of 80–85%) at all the stages. All the indexes for the transgenic and WT plants were determined in flag leaves following the soil water measurements. Relative electrolyte leakage (REL) was measured on the basis of the relative conductivity of the leaves as previously described by Zhang and Huang (2010). MDA content was determined as previously described by Jan *et al.* (2017). Relative water content (RWC) was measured using fresh leaves as previously described by Ma *et al.* (2017). Total soluble protein was measured using Coomassie Brilliant Blue according to standard methods (Bradford, 1976). The proline content of fresh leaves was determined as previously described by Hosseini *et al.* (2017), and the total chlorophyll content of fresh, fully expanded leaves was also determined according to Hosseini *et al.* (2017). The net photosynthetic rate of the flag leaves of 10 randomly selected plants was measured using a LI-6400XT photosynthesis system (LI-COR) in full sunlight (Lang *et al.*, 2017). All measurements were conducted in the morning between 09.00–11.00 on clear days.

### Measurement of soluble protein content and glutamine synthetase activity in leaves

Twenty plants were randomly selected 1 week after flowering at the Shijiazhuang field site in 2012, and the flag and next (second) leaf down the stem were collected for determination of the soluble protein content. Glutamine synthetase (GS) activity was also determined, as previously described (Rhodes *et al.*, 1975; Pécsváradi *et al.*, 2009).

### Proteomic analysis

Flag leaves of plants from the T349 line and the WT (Jimai19 genetic background) were harvested from drought-treated plants at 1 week after flowering. The leaves were frozen in liquid nitrogen and stored at –80 °C. Proteomic analysis consisted of protein extraction, electrophoresis and image analysis, in-gel tryptic digestion, MALDI-TOF/TOF MS analysis, and database searching, all using methods previously described by Jiang *et al.* (2008, 2014). The experiment was replicated three times.

### Root system analysis

#### Hydroponic experiment

In order to observe the characteristics of the root systems, plants of the T349 and T398 transgenic lines and the WT (Jimai19 genetic background) were cultured in Hoagland medium with 18% PEG-6000. The hydroponic culture was carried out in a growth chamber with the following conditions: a 16/8 h day night cycle of 300 µmol m^–2^ s^–1^ at 20 (±1) °C, 50–70% relative humidity. At 7 d after germination, the seedlings were transferred to plastic pots containing 1 l of nutrient solution and grown for 2 weeks; the solution was refreshed every 2 d. Details of the nutrient solution are given in Ren *et al*, (2012) and it was supplemented with 18% PEG for the stress treatment. After 2 weeks, 20 plants from each line were harvested to measure the root fresh weight, dry weight, and root diameter. For microscopic observation of the root system during the seedling stage, the maturation regions of each line were fixed with FAA solution (3.7% formaldehyde, 0.5% acetic acid, 50% ethanol), and then washed three times with 70% ethanol (10 min each). All plants were dehydrated in a graded ethanol series (30%, 50%, 70%, 80%, 95%, 100%) followed by three incubations in 100% ethanol. The dehydrated samples were then treated twice with propylene oxide as a transitional fluid for 30 min each, and they were then embedded in Spurr’s Resin. Ultrathin sections (~1 µm thick) were cut using a diamond knife in an ultramicrotome (MT-X; RMC). The sections were stained with 1% Toluidine Blue for 1 min, washed, and photographed using an Olympus light microscope. The experiment was conducted three times independently.

#### Root tube experiment

In the 2012–2013 growing season, seedlings of transgenic and WT at the two-leaf stage were planted in root tubes in order to examine root characteristics during the whole growth process under a rainproof shelter. Each root tube (25 cm diameter, 240 cm length) was filled with 26.6 kg of topsoil from the field and was irrigated by an amount calculated to maintain maximum field moisture capacity (28%). Controls were watered three times (before planting, at the jointing stage, and at the flowering stage) and the droughted treatments received water only before planting. Each treatment was replicated five times, from which the three most similar tubes were selected for analysis. Root length, surface area, and biomass were measured at the jointing and flowering stages in the following soil layers: 0–40, 40–80, 80–120, 120–160, 160–200, and 200–240 cm. The roots were analysed using a WinRHIZO root-scanning analysis system. The activity of the roots in the different soil layers was measured as α-Naphthylamine oxidation during the grain-filling stage, as described by Liu *et al.* (2014), and is indicative of the vigor of roots.

### RNA-seq

Plants of the transgenic line T349 and the WT (Jimai19 genetic background) were examined using RNA-seq analysis. Seedlings (1 week after germination) were grown for 14 d under hydroponic conditions and were then exposed to air for 5 h drought. Three replicates were used. The RNA-seq analysis was completed by the OE Biotech company (Shanghai, China). Total RNA was extracted using a mirVana miRNA Isolation Kit (Ambion) following the manufacturer’s protocol. RNA integrity was evaluated using a 2100 Bioanalyzer (Agilent Technologies). Samples with an RNA Integrity Number (RIN) ≥7 were selected for subsequent analysis. The libraries were constructed using a TruSeq Stranded mRNA LT Sample Prep Kit (Illumina) according to the manufacturer’ s instructions. The libraries were sequenced on either a HiSeq 2500 or HiSeq X Ten sequencing platform (Illumina) and paired-end reads of 125–150 bp were generated. Differentially expressed genes were identified using the DESeq R package functions estimateSizeFactors and nbinomTest. A *P*-value <0.05 and a fold-change >2 were set as the thresholds for significantly differential expression. Gene Ontology (GO) enrichment analyses of DEGs were performed using R based on the hypergeometric distribution.

### qPCR

Total RNA from flag leaves and roots of plants 1 week after flowering was extracted using Trizol reagent (Takara), and cDNAs were synthesized using a PrimeScript First-Strand cDNA Synthesis kit (Takara) according to the manufacturer’s protocol. qPCR was performed using SYBR master mix (Tiangen) with an ABI Prism 7500 real-time PCR system (Lifetech) as previously described (Yu *et al.*, 2017). The relative transcript levels of genes were calculated using the 2^−ΔΔ*C*T^ method (Li *et al.*, 2011). All primers used for qPCR are given in Supplementary Table S15.

### Measurement of melatonin content

Plants were cultured in Hoagland medium with or without 18% PEG-6000. After 7 d, fresh tissues were ground in liquid nitrogen and a 9× sample volume of PBS buffer (PH 7.4) was then added. The homogenate was centrifuged at 8000 *g* for 30 min at 4 °C. The supernatant was then used for measurements. The melatonin content of leaves and roots was determined using a plant melatonin ELISA kit (J&L Biotechnology, USA).

### Effects of melatonin on the drought tolerance of transgenic wheat

Seeds from each of the transgenic and WT lines were divided into three treatments with three repeats for each (100 seeds per repeat). Seeds were placed in a culture dish with two layers of filter paper and were then treated with 5 ml of solution containing water and 18% PEG, or 18% PEG with 200 µM melatonin. After 7 d, the shoot height, total root length, shoot fresh weight, and root fresh weight were measured.

### Statistical analysis

Data for morphology, physiology, agronomic traits, and yield were analysed by one-way ANOVA using the SPSS 16.0 statistical software. The volume changes of protein spots were analysed using Student’s *t*-test.

## Results

### Overexpression of the soybean GmDREB1 transcription factor in wheat improves growth under drought stress

After genetic transformation of two popular wheat varieties (Jimai19 and Jimai20) using the particle bombardment method, T_0_–T_3_ generation transgenic plants were genotyped using PCR (Supplementary Table S1). Our aim was to test the drought-related utility of *GmDREB1* expression in wheat plants in the field, and we were also interested in exploring the potential crop physiological and molecular mechanisms related to enhanced drought resistance of the transgenic plants. We therefore performed three analyses: (1) yield comparisons and examination of physiological characteristics in the field with controlled irrigation conditions; (2) examination of physiological characteristics of plants grown in pots under a controllable rainproof shelter; and (3) extensive examination of drought resistance of the transgenic plants at the seedling stage in the greenhouse.

**Fig. 1.**
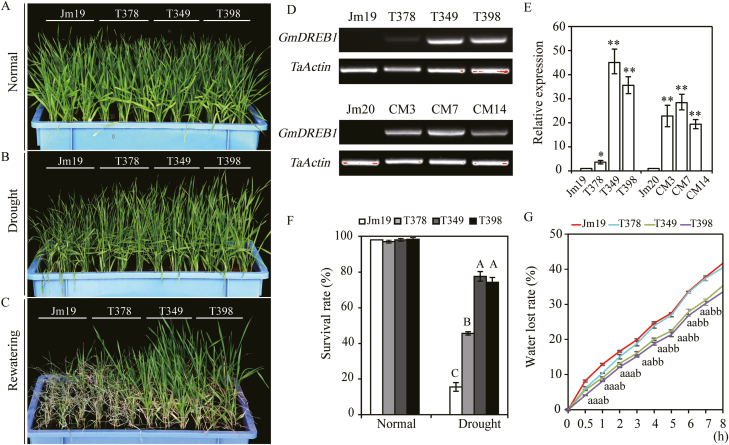
Overexpression of *GmDREB1* in wheat improves growth under drought stress. Seedlings were grown to the three-leaf stage in soil with sufficient water, after which water was withheld for 21 d, followed by rewatering for 7 d. (A) Seedlings of the wild-type Jimai19 (Jm19) and three transgenic lines after 7 d of growth under well-watered (‘Normal’) conditions, and (B) seedlings after 7 d of water being withheld. (C) Seedlings after 7 d of rewatering. (D) RT-PCR for *GmDREB1* expression in the wild-types Jimai19 (Jm19) and Jimai20 (Jm20) and their respective transgenic lines. (E) qPCR analysis of the expression level of *GmDREB1*. Significant differences between wild-type and the corresponding transgenic lines were determined using Student’s *t*-test: *P<0.05; **P<0.01. (F) The survival rates of wheat seedlings as determined following 7 d of rewatering. Different letters indicate significant differences between means within a treatment as determined by ANOVA (*P<0.01). (G) Rate of water loss of detached leaves of seedlings at the three-leaf stage as determined over an 8-h period. Different letters indicate significant differences between means within a time-point as determined by ANOVA (*P<0.05). In each case, the order of the letters corresponds to the order of the genotypes shown in the key above the graph. All data represent means (±SE) of four replicates.

To evaluate and select drought-tolerant transgenic wheat lines, initial tests were conducted in the greenhouse. The survival rate of the transgenic line T349 was significantly higher than that of the WT (Jimai19) under drought conditions (Supplementary Fig. S2). Based on their survival rates and drought-tolerant phenotypes, six transgenic lines were selected for further study, namely T349, T398, and T378 (Jimai19), and CM3, CM7, and CM14 (Jimai20). Semi-quantitative RT-PCR showed that *GmDREB1* was effectively transcribed in the T_4_ generation lines (Fig. 1D), and Southern blotting further confirmed that two copies of the gene had been integrated into the genomes of four of the transgenic lines (T349, T398, CM7, and CM14; Supplementary Fig. S3).

To characterize the drought-tolerance effects resulting from the overexpression of *GmDREB1* at the seedling stage, we examined three transgenic lines under greenhouse conditions: T349, T398, and T378, all in the Jimai19 genetic background (Fig. 1A–C). After water was withheld for 7 d, WT plants showed obvious drought stress symptoms such as leaf rolling. In comparison, leaf rolling in T349 and T398 was very slight (Fig. 1B) and, importantly, no differences were observed when plants were grown under normal conditions (Fig. 1A). After rewatering following the drought treatment, the survival rates of the T378, T349, and T398 lines (45.57%, 77.53%, and 74.17%, respectively) were significantly higher than that of the WT Jimai19 (15.57%; *P*<0.01, Fig. 1C, F). Leaves of all the transgenic lines had lower rates of water loss than those of the WT during the first 2 h of drought stress, after which the rates remained significantly lower in the T349 and T398 lines (*P*<0.01, Fig. 1G), whilst the rate in T378 was comparable to that in the WT.

Overexpression of *GmDREB1* thus enhanced the drought-stress tolerance of the transgenic lines. Specifically, we found that the tolerance of the T349 and T398 lines was superior to that of T378 and the WT, and this was consistent with the expression levels of *GmDREB1*, with higher expression being associated with greater drought tolerance (Fig. 1D, E).

### *GmDREB1* transgenic wheat exhibits increased yield and improved performance for multiple traits in the field

**Fig. 2.**
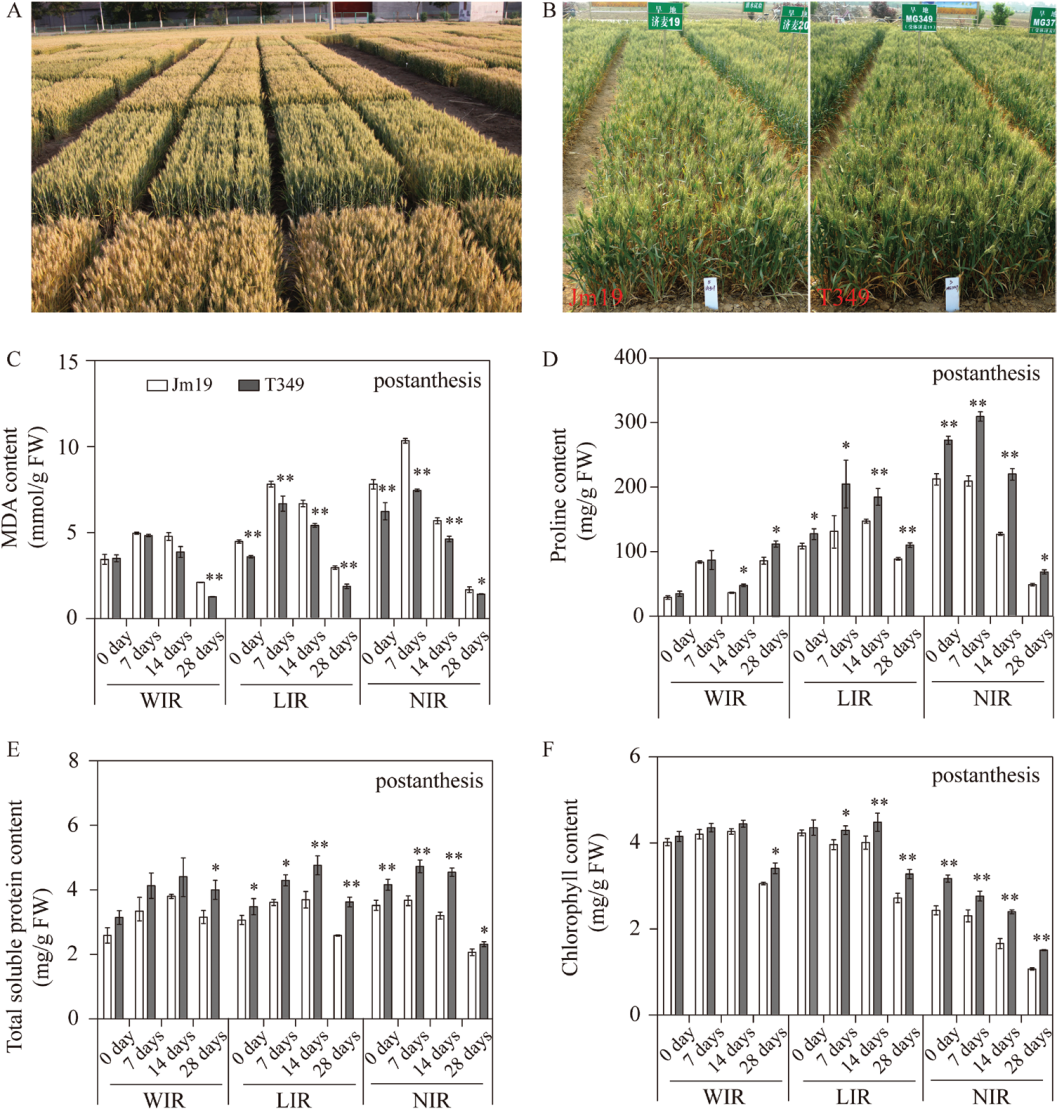
*GmDREB1* transgenic wheat exhibits improvements in a variety of physiological traits. (A) Examples of plots of drought tolerance studies conducted in the field. (B) Field performance of the transgenic line T349 and the wild-type Jimai19 (Jm19) during the 2012 growing season at the Shijiazhuang site under limited-irrigated conditions. (C–F) Physiological traits of the lines were analysed in flag leaves after flowering in field experiments under well-irrigated (WIR), limited irrigation (LIR), and no irrigation (NIR) conditions at the Shijiazhuang site in the 2012 growing season. (C) Malondialdehyde (MDA) content, (D) proline content, (E) total soluble protein content, and (F) chlorophyll content. Data are means (±SE) of three replicates. Significant differences between the wild-type and the transgenic plants at each time-point were determined using Student’s *t*-test: *P<0.05; **P<0.01.

Having established that transgenic expression of *GmDREB1* increased drought tolerance under greenhouse conditions, we used T_3_ generation plants to evaluate yield performance under drought conditions in the field. In addition, we also examined phenotypes under rainproof shelters (Fig. 2A, Supplementary Fig. S1). Experiments were conducted in three years (2011–2013) at three sites in the main wheat production area of China. The results showed that the grain yields of the transgenic lines were significantly higher than the WT for both the Jimai19 and Jimai20 genetic backgrounds under non-irrigated (NIR) and limited-irrigation (LIR) conditions (*P*<0.05; Table 1). Under well-irrigated (WIR) conditions, the yields of the transgenic lines were not significantly different from those of the WT.

**Table 1.** Grain yields of the wild-type and *GmDREB1* transgenic lines averaged across the three growing seasons at the three experimental sites

Lines	Shijiazhuang			Jinan		Linfen	
	NIR	LIR	WIR	LIR	WIR	LIR	WIR
T349	4913.83±126.48^a^	6249.64±207.00^a^	7463.99±157.21^a^	5532.57±152.66^A^	6865.25±133.19^a^	6830.17±119.06^A^	7692.58±194.31^a^
T398	4802.71±37.55^a^	6187.34±149.11^a^	7332.18±131.66^a^	5509.74±177.34^A^	6840.24±145.91^a^	6605.40±109.32^B^	7631.63±121.49^a^
Jimai19	4367.50±163.94^b^	5614.65±172.84^b^	7150.96±127.12^a^	4671.76±147.08^B^	6359.66±211.25^a^	6303.47±102.06^B^	7466.27±164.92^a^
CM3	4927.29±120.54^a^	6078.70±245.63^a^	7348.29±122.34^a^	4580.15±90.47^a^	7117.15±126.78^a^	6957.68±135.41^a^	7706.10±72.79^a^
CM7	4846.64±71.13^a^	6289.78±228.40^a^	7599.54±88.17^a^	4667.88±106.39^a^	6790.19±159.38^a^	6676.63±149.94^a^	7325.45±140.43^a^
CM14	5064.55±76.19^a^	6231.62±149.25^a^	7321.92±68.40^a^	4581.90±82.61^a^	6843.12±153.30^a^	6540.70±101.13^ab^	7345.35±216.11^a^
Jimai20	4727.22±80.95^a^	5767.12±204.13^b^	7304.86±118.49^a^	4089.50±53.74^b^	6828.69±107.12^a^	6206.90±68.39^b^	7271.43±202.52^a^

Data are grain yields in kg ha^–1^ and represent the mean values (±SE) obtained at each of the experimental sites across the three growing seasons 2011–2013 (i.e. *n*=3). Yields for individual years are given in Supplementary Table S2. NIR, non-irrigated; LIR, limited irrigation; WIR well-irrigated. Different letters within a treatment and genotype at an individual site indicate significant differences as determined by ANOVA, at *P*<0.05 (lowercase letters) or *P*<0.01 (uppercase letters)

At the Shijiazhuang site in the 2011–2013 growing seasons, the yields of the T349 and T398 lines were significantly increased compared to WT Jimai19 by 9.96–12.51% and 10.20–11.31% under the NIR and LIR treatments, respectively (*P*<0.05). In the 2012–2013 growing season, the yields of the CM3, CM7, and CM14 lines were significantly increased compared to WT Jimai20 by 2.53–7.14% and 5.40–9.06% under the NIR and LIR conditions, respectively (*P*<0.05; Table 1, Supplementary Table S2). At the Jinan site in the 2011–2013 growing seasons, the yields of the T349 and T398 lines were significantly increased by 17.94–18.43% and those of the CM3, CM7, and CM14 lines were significantly increased by 12.00–14.14% compared to the respective WT plants when grown under LIR conditions (Table 1, Supplementary Table S2). At the Linfen site under LIR conditions, the yields of the T349 and T398 lines in the 2011–2013 growing season were significantly increased by 4.79–8.36% compared to the WT, and those of the CM3, CM7, and CM14 lines in the 2012–2013 growing season were significantly increased by 5.38–12.10% compared to the WT (*P*<0.05) (Table 1, Supplementary Table S2).

We examined three major yield components and found that the most obvious difference was a significant increase in the number of spikes in the transgenic lines compared with the WT when grown under LIR conditions (*P*<0.05) (Fig. 2B, Supplementary Fig. S4, Supplementary Table S3). Values for thousand grain weight (TGW) were highly variable across the three experimental sites (Supplementary Table S3): the TGWs of T349 and T398 were significantly higher than those of the WT at all three sites, while those of the CM3 and CM14 lines were only significantly higher than the WT at the Shijiazhuang site (*P*<0.05). In the 2013 growing season, we also determined the harvest index at the Shijiazhuang site for the T349 line and found that there was no significant difference compared with the WT (Supplementary Table S4). Under LIR conditions, the rate of increase in biomass of the T349 line was not significantly different to that of the WT (Supplementary Table S4; Flowering–Jointing, g plant^–1^); however, after flowering, the biomass of T349 increased significantly compared with the WT (Mature–Flowering). At harvest, the grain yield was significantly higher than that of the WT.

It is known that drought stress after flowering has a significant impact on yield in wheat (Guoth *et al.*, 2009; Shukla *et al.*, 2015), and we therefore examined physiological traits 2 weeks after flowering during the 2011–2012 growing season at the Shijiazhuang site (Fig. 2C–F). It was notable that, compared to WT plants under both LIR and NIR conditions, the transgenic lines had significantly lower MDA content (Fig. 2C) and significantly higher levels of proline (Fig. 2D), total soluble proteins (Fig. 2E), and chlorophyll (Fig. 2F) (*P*<0.01). We also examined the T349 and T398 lines grown under rainproof shelters, and found that under drought stress the relative electrolyte leakage (REL) was significantly lower than that of the WT at each stage from jointing through to grain-filling (*P*<0.05; Supplementary Fig. S5A). It was notable that the extent of the difference also gradually increased with time. The MDA content of WT plants was significantly higher than that of the transgenic lines during most growth stages, with the exception of the jointing stage (*P*<0.05) (Supplementary Fig. S5B). The RWC of the transgenic lines was only significantly higher that the WT plants during the grain-filling period (*P*<0.05) (Supplementary Fig. S5C). The proline content of the in transgenic lines was significantly higher than that of the WT plants consistently at all growth stages (*P*<0.05) (Supplementary Fig. S5D) and similar results were also seen for total soluble protein content (Fig. S5E). Under non-drought conditions, the proline content and REL exhibited no significant differences between the WT and transgenic lines during the grain-filling stage. Collectively, the results from the field and rainproof-shelter experiments established that transgenic plants expressing *GmDREB1* exhibited improved drought tolerance, most likely resulting from alterations in their osmotic metabolism.

**Fig. 3.**
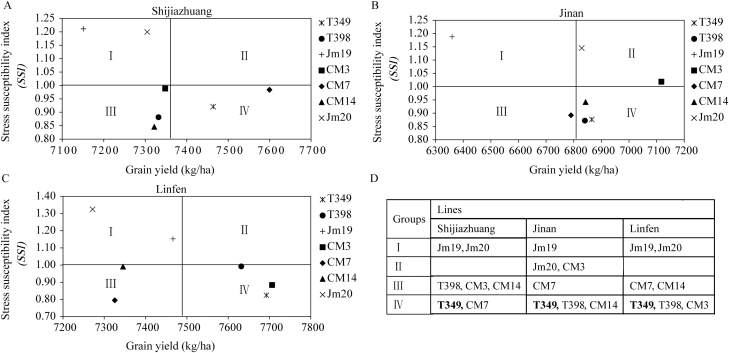
Cluster analysis of the drought tolerance of transgenic wheat overexpressing *GmDREB1*. The stress susceptibility index is plotted against the grain yield under well-irrigated conditions for the Jimai19 (Jm19) and Jimai20 (Jm20) wild-types and their respective transgenic lines at the three experimental sites: (A) Shijiazhuang; (B) Jinan; and (C) Linfen. (D) The genotypes were clustered into the following groups: I, low yield and low drought resistance (high SSI); II, high yield and low drought resistance; III, low yield and high drought resistance (low SSI); and IV, high yield and high drought resistance.

The stress susceptibility index (SSI) is used extensively as a method for distinguishing the stress-tolerance performance between cultivars, with low SSI values being indicative of stress tolerance (Sio-Se Mardeh *et al.*, 2006). We found that the SSI values of all of the transgenic lines were significantly lower than those of the WTs, for all growth conditions at all three of the experimental sites (*P*<0.05; Table 2). We also conducted cluster analysis using SSI values and grain yield under WIR conditions, as previously described (Yu *et al.*, 2017), and found that the WT plants were clustered into groups I or II (high SSI) whereas the transgenic lines were clustered into groups III or IV (low SSI), suggesting that the transgenic plants had greater drought tolerance and higher yield potentials across the different environments (Fig. 3). We examined the grain yield responses of individual genotypes to the environmental index (Supplementary Fig. S6), and this clearly indicated the advantage of the transgenic lines over WT plants across all the evaluated environments. We also examined grain yield responses in relation to spikes m^–2^, grains per spike, and TGW (Supplementary Fig. S7), and these showed consistent trends between the two genetic backgrounds (Jimai19 and Jimai20).

**Table 2.** Mean stress susceptibility index for yield at each experimental site across the three growing seasons

Lines	Site			Mean
	Shijiazhuang	Jinan	Linfen	
T349	0.92^a^	0.88^a^	0.82^a^	0.87^a^
T398	0.88^a^	0.87^a^	0.99^b^	0.92^a^
Jimai19	1.21^b^	1.19^b^	1.15^c^	1.18^b^
CM3	0.99^a^	1.02^a^	0.88^a^	0.96^a^
CM7	0.98^a^	0.89^a^	0.79^a^	0.89^a^
CM14	0.85^a^	0.94^a^	0.99^a^	0.93^a^
Jimai20	1.20^b^	1.15 ^b^	1.32^b^	1.22^b^

Data are means of three replicates (years). Different letters within a site and genotype indicate significant differences as determined by ANOVA (P<0.05).

We examined water-use efficiency (WUE) at the Shijiazhuang site and found that values for the transgenic plants (T349=18.91 kg ha^–1^ mm^-1^; T398=18.75 kg ha^–1^ mm^-1^) were significantly higher than that of the WT plants (Jm19=15.70 kg ha^–1^ mm^-1^; *P*<0.05), averaging a 20% increase in this trait. Note that rainfall at the three sites was very limited: a survey at the sites from 2010 to 2013 (Supplementary Fig. S11) showed that soil moisture content in the 0–40 cm soil layer in the field remained at 10–18%, which is equivalent to moderate drought stress (Supplementary Table S12).

### Transgenic plants expressing *GmDREB1* have enhanced photosynthesis resulting from increased chloroplast stability

**Fig. 4.**
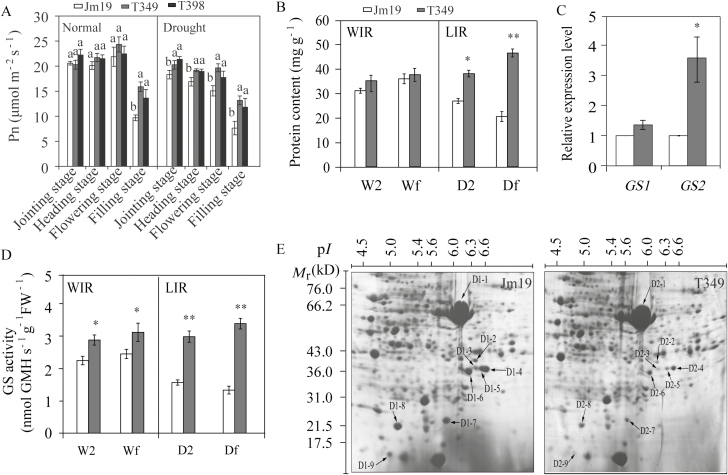
Photosynthetic parameters in the T349 and T398 transgenic wheat lines overexpressing *GmDREB1* compared with the wild-type Jimai19 (Jm19). Leaves were harvested from plants subjected to limited irrigation at 7 d after flowering in the 2012 growing season at the Shijiazhuang site (A) Net photosynthesis rate (Pn) under normal (well-watered) conditions and under drought stress (rainproof shelter). Different letters indicate significant differences between means as determined by ANOVA (*P*<0.05). (B) Total soluble protein content of leaves of plants grown in the field under well-irrigated (WIR) and limited irrigation (LIR) conditions. W2 and Wf are the second leaf from the top of the stem and the flag leaf, respectively, in the WIR treatment; D2 and Df are the second leaf from the top of the stem and the flag leaf, respectively, in the LIR treatment. (C) Relative expression levels of *glutamine synthetase 1* (*GS1*) and *GS2* in the flag leaf. (D) GS activity under the WIR and LIR treatments. In (B–D) significant differences were determined using Student’s *t*-test: *P<0.05; **P<0.01. All data are means (±SE) of three replicates. (E) Two-dimensional electrophoresis of proteins from the flag leaves of Jimai19 and T349 under the LIR treatment. The protein spots correspond to those listed in Table 3.

Drought stress is known to reduce photosynthesis, which subsequently decreases leaf expansion, impairs the photosynthetic machinery *per se*, and causes premature leaf senescence (Farooq *et al.*, 2010). Rubisco is a major target for improving crop photosynthesis and yields (Prins *et al.*, 2016). Glutamine synthetase activity and expression are known to be significantly modulated by drought stress (James *et al.*, 2018). In light of our observations of increased grain yields under drought stress in the transgenic lines grown in the field (Table 1, Supplementary Table S2), we conducted additional analysis of photosynthesis. We found that the chlorophyll content of the WT was significantly decreased compared to the transgenic lines under both LIR and NIR conditions, and both in the field (*P*<0.01) and under rainproof shelters under drought stress (*P*<0.05; Fig. 2F, Supplementary Fig. S5F). The photosynthetic efficiency of the transgenic plants was significantly higher than that of the WT over the whole growth period under drought stress under the rainproof shelter (*P*<0.05; Fig. 4A).

We compared the total protein content after the flowering stage in drought-stressed T349 and WT plants (Jimai19 background) grown in the field using a two-dimensional electrophoresis assay and observed significantly different signals for nine protein gel spots. Following selection, digestion, and MS-based identification of peptides, amino acid sequence analysis in NCBI revealed that each of these nine spots contained proteins from the large subunit of Rubisco (Table 3). Specifically, spot D-1 was a Rubisco large subunit and spots D-2 to D-9 were degraded products of the large subunit. This indicated that Rubisco degradation occurred significantly more slowly in T349 than in the WT, which suggested that the photosynthetic efficiency of the transgenic plants might be higher under drought conditions. Glutamine synthetase (GS), a key enzyme in plant nitrogen metabolism, is essential for both primary assimilation and recycling (Ferreira *et al.*, 2017). We found that the expression level of *GS2* was signiﬁcantly higher in T349 than in the WT (*P*<0.05; (Fig. 4C). In addition, both the total soluble protein content and the extent of GS activity in the flag leaf and the next leaf on the stem were higher in T349 than in WT plants under drought stress (*P*<0.05; ) (Fig. 4B, D). These results indicated that transgenic plants expressing *GmDREB1* displayed enhanced photosynthesis, which was apparently the result of an increase in the stability of the chloroplasts under drought stress.

**Table 3.** Identification of differentially expressed proteins between drought-stressed wild-type Jimai19 and transgenic T349 plants as determined by amino acid sequence analysis in the NCBI database

Protein spot no. on gel*	Identified protein name	Species	GenInfo Identifier (gi)	Molecular weight (kDa)	Isoelectric point	Fold-change^†^
D-1	Rubisco large subunit	*Triticum aestivum*	12344	47.6	6.60	1.70
D-2	Rubisco large subunit	*Cremaspora triflora*	1770202	52.8	6.44	–3.00
D-3	Rubisco large subunit	*Haworthia cymbiformis*	37361619	47.0	6.18	–2.33
D-4	Rubisco large subunit	*Aglaonema nitidum*	62420476	53.4	6.13	–2.69
D-5	Rubisco large subunit	*Aglaonema nitidum*	62420476	53.4	6.13	–2.91
D-6	Rubisco large subunit	*Anemone rupicola*	296033300	53.8	6.33	–2.84
D-7	Rubisco large subunit	*Cyrtochilum myanthum*	220683351	46.8	6.21	–2.43
D-8	Rubisco large subunit	*Elytrigia repens*	342196494	21.1	5.76	–2.72
D-9	Rubisco large subunit	*Triticum aestivum*	12344	47.6	6.60	–2.59

* The protein spots are shown in Fig. 4E.

^†^ Fold-change: positive values represent the fold-change of up-regulation in T349 compared to Jimai19; negative values represent the fold-change of down-regulation in T349 compared to Jimai19.

### Improved drought tolerance in *GmDREB1* transgenic lines is related to changes in melatonin synthesis

Given the increased grain yields in the transgenic lines grown in the field, and in light of the observed differences in physiological traits in both the field and rainproof-shelter experiments, we next conducted experiments in the greenhouse in order to examine morphological changes in detail and to investigate the molecular mechanism(s) that influence the enhanced drought tolerance of the transgenic plants. We grew seedlings hydroponically and found that the root fresh and dry weights of the T349 and T398 transgenic lines were significantly greater than those of the WT plants under drought stress (*P*<0.05; Supplementary Fig. S9). We also found that the mean root and stele diameters of the transgenic plants under drought stress conditions were significantly larger than those of the WT plants (*P*<0.05). In a separate experiment, we grew plants in root tubes and examined them at different depths. We found that across the full depth of the tubes (0–240 cm) there were no significant differences in the root traits between the transgenic and WT plants at the jointing stage (Supplementary Table S5). During the flowering stage, the root surface area of the transgenic plants was significantly greater than that of WT under drought stress, and the total root length was significantly greater than that of WT under both drought and normal conditions; there was a 24.58% (*P*<0.01) increase in the root dry weight of the transgenic plants compared to the WT under drought stress (Supplementary Table S5). Transgenic plants clearly had more root tissue than the WT in deeper layers under drought stress (Supplementary Tables S6, S7).We also determined root activity (α-Naphthylamine oxidation, an indicator of the vigor of the roots) and found that the activity of the roots of transgenic plants in the deeper layers (>40 cm) was significantly higher than that of the WT plants drought stress (*P*<0.01; Supplementary Fig. S10A). Under non-drought conditions, the root activity of the transgenic plants in the 0–40 cm layer was not different to that of the WT. For the 40–80 cm layer, there was an obvious increase for the transgenic roots (Supplementary Fig. S10B). Such root characteristics are very likely to be beneficial for wheat to absorb deep water under drought conditions after the jointing stage.

**Fig. 5.**
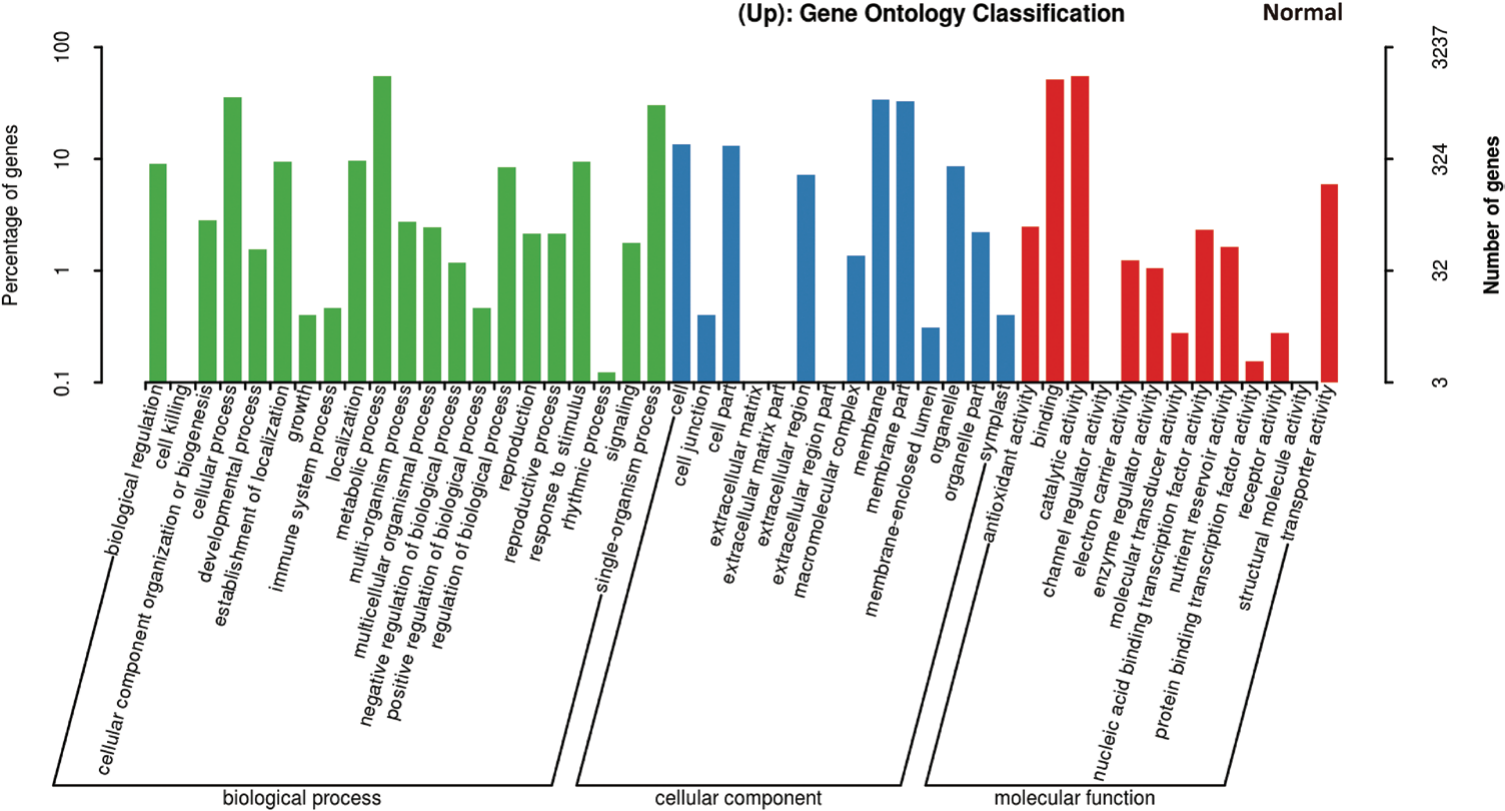
Gene Ontology (GO) analysis in the T349 transgenic wheat line overexpressing *GmDREB1* compared with the wild-type Jimai19 (Jm19). Seedlings at 7 d after germination were transferred to hydroponic culture for 14 d and grown normally (i.e. no stress was imposed). Samples (three biological replicates) were then subjected to RNA-seq analysis and genes up-regulated in the transgenic line were identified and used for the GO analysis.

**Fig. 6.**
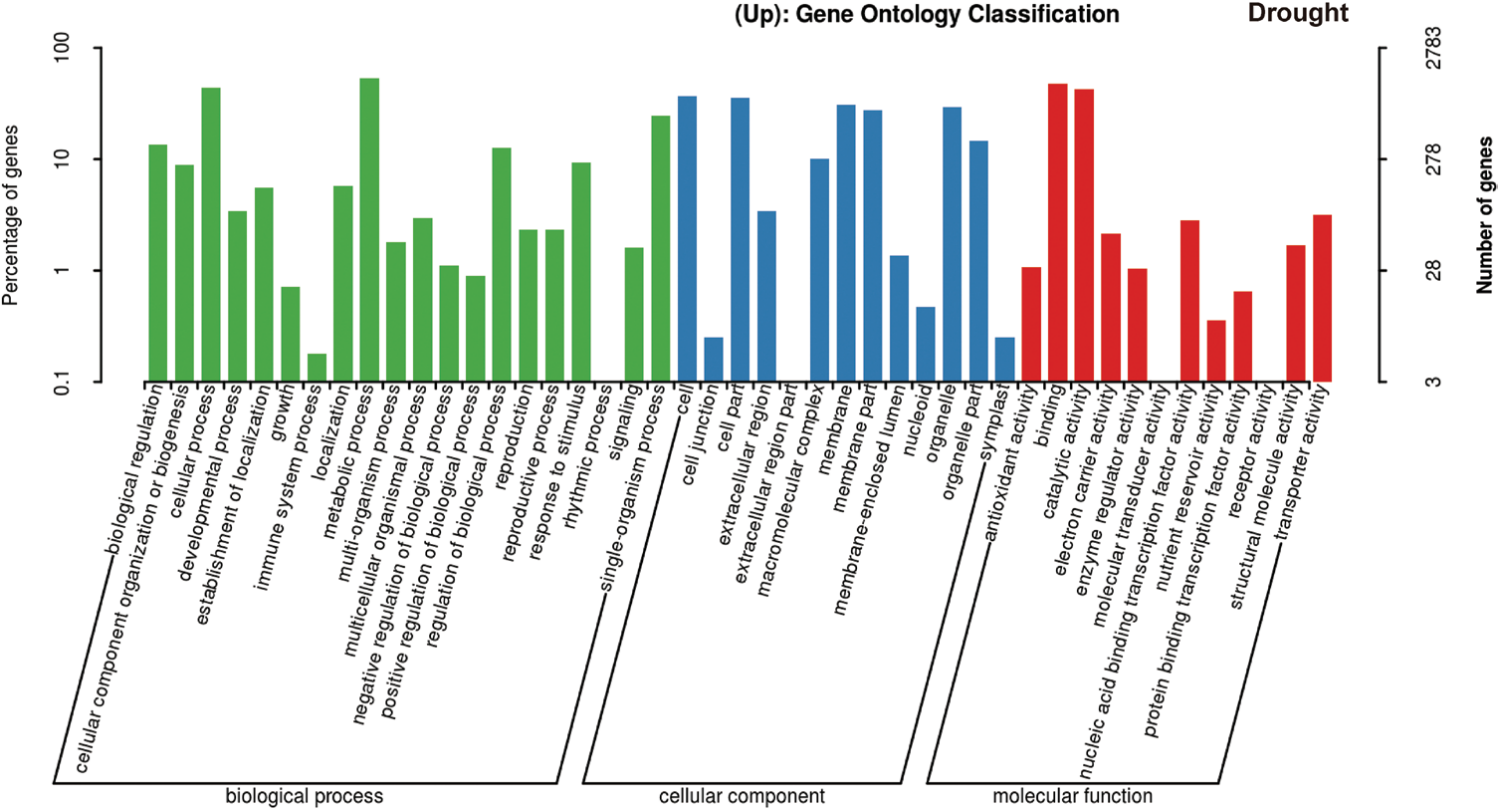
Gene Ontology (GO) analysis in the T349 transgenic wheat line overexpressing *GmDREB1* compared with the wild-type Jimai19 (Jm19). Seedlings at 7 d after germination were transferred to hydroponic culture for 14 d and were then exposed to air 5 h for water stress Samples (three biological replicates) were then subjected to RNA-seq analysis and genes up-regulated in the transgenic line were identified and used for the GO analysis.

**Fig. 7.**
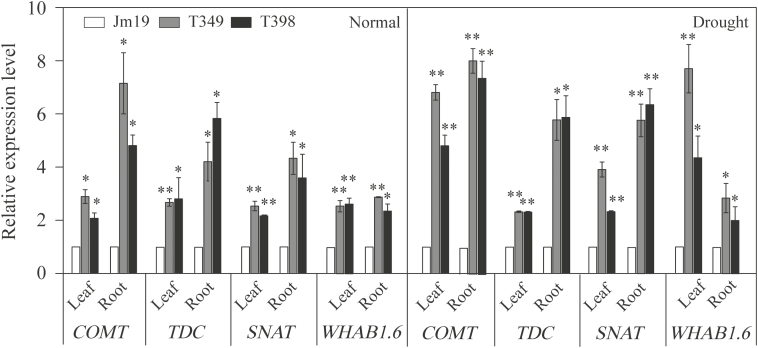
qPCR analysis of expression levels of downstream genes in leaves and roots of the T349 and T398 transgenic wheat lines overexpressing *GmDREB1* compared with the wild-type Jimai19 (Jm19) under well-watered and drought treatments. Seedlings at 7 d after germination were transferred to hydroponic culture for 14 d and were then exposed to the 5 h for water stress. Total RNA of leaves and roots were extracted. Expression is relative to that of the wild-type, the value of which was set as 1. *COMT*, caffeic acid O-methyltransferase; *TDC*, tryptophan decarboxylase; *SNAT*, serotonin N-acetyltransferase; *WHAB1.6*, chlorophyll a-b binding protein. Data are means (±SE) of four replicates. Significant differences between the wild-type and each transgenic line were determined using Student’s *t*-test: *P< 0.05; **P<0.01.

Given that *GmDREB1* is a transfer factor, we next conducted RNA-seq analysis to identify its downstream targets. Comparing T349 with the WT (Jimai19), a total of 2509 and 3267 differentially expressed genes (DEGs) were up-regulated under normal and drought conditions, respectively (Supplementary Tables S8, S9), and 1537 and 2047 DEGS were down-regulated under normal and drought conditions, respectively. GO analysis indicated that some of the up-regulated genes were clustered into stress response-related terms such as ‘response to stimulus’ and ‘signaling’ (Figs. 5, 6; Supplementary Tables S10, S11). In particular, the GO analysis indicated that some *COMT* (caffeic acid O-methyltransferase; TRIAE_CS42_7BL_TGACv1_579947_AA1911100) and *COMT-like* genes (TRIAE_CS42_7DL_TGACv1_603606_AA1986230, TRIAE_CS42_7AL_TGACv1_558347_AA1792640) belonging to the GO term ‘O-methyltransferase activity’ were up-regulated. The hormone melatonin is synthesized from tryptophan via a series of enzymes including tryptophan decarboxylase (TDC), tryptamine 5-hydroxylase (T5H), serotonin N-acetyltransferase (SNAT), and COMT, with the latter being responsible for last step of biosynthesis (Lee *et al.*, 2014). Our RNA-seq data showed that *TDC* and *SNAT*, which encode two additional melatonin biosynthesis-related proteins, were also up-regulated in the T349 transgenic line (Supplementary Tables S8, S9). qPCR analysis confirmed that *COMT*, *TDC* (TRIAE_CS42_4AL_TGACv1_288392_AA0947370), and *SNAT* (TRIAE_CS42_7BL_TGACv1_579238_AA1905680) were indeed up-regulated in leaves and roots of transgenic plants relative to the WT under both normal and drought-stress conditions (Fig. 7).

The expression levels of *COMT*, *TDC*, and *SNAT* in roots were higher than those in leaves, and were also higher under drought stress than under normal conditions, with particularly pronounced differences evident for *COMT* (Fig. 7). In addition, both the RNA-seq and qPCR analyses showed that *WHAB1.6* (TRIAE_CS42_7AS_TGACv1_570660_AA1839080), encoding a chlorophyll *a*/*b*-binding protein (Lamppa *et al.*, 1985), was also significantly up-regulated by drought stress in both the roots and leaves of the transgenic plants.

**Fig. 8.**
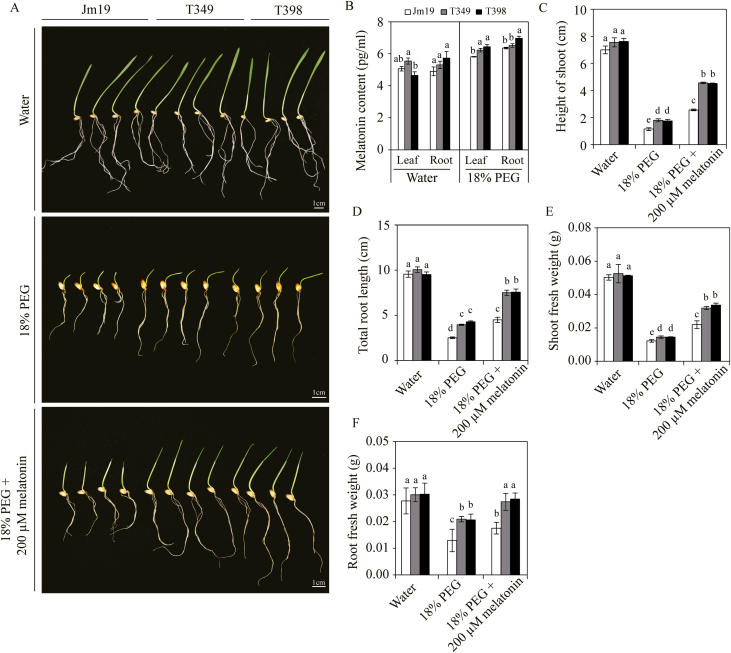
Melatonin improves drought tolerance in transgenic wheat overexpressing *GmDREB1*. Seeds of the wild-type Jimai19 (Jm19) and the T349 and T398 transgenic lines were grown hydroponically in water, or in water plus 18% PEG or plus 18% PEG and plus 200 µM melatonin. After 7 d, the shoot height and fresh weight, and the total root length and fresh weight were determined. (A) Images of the plants under the different treatments. (B) Leaf and root melatonin contents for plants grown in water or 18% PEG. (C) Shoot height, (D) total root length, (E) shoot fresh weight, and (F) root fresh weight for plants grown under the three different treatments. Data represent means (±SE) of three replicates, with each replicate consisting of 100 plants. Different letters indicate significant differences between genotypes within treatments as determined using ANOVA (*P*<0.05).

To determine whether biochemical/metabolic effects corresponded to the results of the molecular genetics analyses, we conducted a hydroponics experiment at the seedling stage and found that the melatonin content in both the roots and leaves of the transgenic lines were significantly increased under water-stress conditions (18% PEG) as compared to the WT (*P*<0.05; Fig. 8B). We also treated transgenic plants with PEG plus melatonin and found that they had significantly longer roots than the WT control plants (Fig. 8A). In addition, the height of the shoots of the transgenic plants increased by 56.4% in T349 and by 81.4% T398 in response to PEG plus melatonin as compared to the PEG-only treatment, and these increases were significantly greater than those observed for the WT (49.0%; Fig. 8C). The shoot fresh weights of the transgenic plants increased by 47.6% in T349 and by 103.2% in T398, which were again significantly greater than the increase observed for the WT plants (32.1%; Fig. 8E). Thus, our results indicated that the increased drought tolerance resulting from the overexpression of *GmDREB1* in transgenic wheat plants under drought stress may be mediated by the phytohormone melatonin.

## Discussion

### *GmDREB1* confers stable drought tolerance to transgenic wheat and improves water-use efficiency in the field

The effects of transgene expression in wheat are known to vary from year to year in the field as influenced by the particular climatic conditions experienced in a given growing season (Bahieldin *et al.*, 2005). Years of rigorous testing in the field are required to characterize the potential benefits of genetic improvement for stress tolerance in crops (Castiglioni *et al.*, 2008). In our study, field testing for three consecutive years at three different locations showed that the yield of two *GmDREB1* transgenic wheat varieties increased by 2.53–18.43% as compared to wild-type (WT) plants under both non-irrigated (NIR) and limited-irrigated (LIR) conditions (Table 1, Supplementary Table S2), but the harvest index did not change significantly between the transgenic lines and the WT. The stress susceptibility index values that we observed highlighted the drought tolerance of the transgenic plants in the different environments (Table 2), thus establishing that the transgenic expression of *GmDREB1* did indeed confer drought tolerance in field-grown wheat, resulting in increased crop yield under drought conditions. A recent study involving a large series of 37 field trials reported that expression of the sunflower transcription factor gene *HaHB4* in wheat resulted in a drought-tolerant phenotype (González *et al.*, 2019). Our results were similar: we found that overexpression of a DREB-like transcription factor from soybean, *GmDREB1*, delayed senescence, improved water-use efficiency, and increased grain yield of transgenic wheat varieties under different field conditions. Both studies therefore show that it is feasible to improve the drought resistance of wheat by transforming a transcription factor gene related to drought resistance from another species. In our study, the yield increases under drought conditions were mainly due to increases in spike number and thousand-grain weight, which was different to the results of González *et al.* (2019) where the increased yield was mainly through an increase in the number of grains per unit area. Our results also showed that *GmDREB1* could confer drought tolerance in wheat by increasing the photosynthetic efficiency, the accumulation of osmoregulation substances, and the synthesis of melatonin. González *et al.* (2019) mention that the improvement of drought tolerance of *HaHB4* transgenic wheat does not depend on *TaDREB1a*, suggesting that *HaHB4* is involved in a different pathway to the DREB-like transcription factor in our *GmDREB1* transgenic plants. This may be the reason why the two genes act through different mechanisms to improve the yield under drought conditions

We examined water-use efficiency (WUE) at the Shijiazhuang site and found that it was significantly higher in the T349 and T398 transgenic plants than in the WT. Expression of *GmDREB1* resulted in deeper and larger root systems (Fig. 8), which would have contributed to the improvement in WUE.

Analysis of the grain yield response of the individual genotypes to the environmental index (Supplementary Fig. S6) and the responses of its components (Supplementary Fig S7, S8) clearly demonstrated the advantage of the transgenic plants over the WT plants across the different environments and between the two varieties. The results clearly indicated that the functions of *GmDREB1* that induce drought tolerance did not depend on the genetic background. We did not find negative phenotypes resulting from the overexpression of *GmDREB1* in wheat, i.e. we observed no differences in traits such as plant height, heading date, or flowering stage between well-watered transgenic and WT plants (Supplementary Tables S13, S14).

It thus appears that the drought tolerance that we observed in plants overexpressing *GmDREB1* is of potentially practical value in breeding efforts aimed at improving wheat performance under drought.

### *GmDREB1* affects the drought tolerance of transgenic wheat by regulating the accumulation of melatonin

In order to determine the regulatory mechanism of the drought tolerance observed in the *GmDREB1* transgenic plants, we identified downstream genes using RNA-seq analysis. We found that *COMT* genes were up-regulated by 2–8-fold in the transgenic lines under drought stress (Fig. 7). *COMT* is responsible for the last step of melatonin biosynthesis, where it methylates *N*-acetylserotonin to produce melatonin (Lee *et al.*, 2014; Shi *et al.*, 2017). In addition, two other melatonin biosynthetic genes, *TDC* and *SNAT*, were also up-regulated in transgenic plants under stress. Under water-stress conditions, the melatonin content in leaves of T349 and T398 plants was significantly higher than that in the WT (Fig. 8B). Furthermore, treatment with melatonin improved the drought tolerance in these transgenic lines, suggesting that melatonin is involved in the effects downstream of *GmDREB1*. Numerous functions of melatonin in plants have been confirmed, and there is strong evidence to suggest that it functions in various stress responses (Shi *et al.*, 2017). Thus, the changes in melatonin synthesis that we observed may be involved in the improved drought tolerance of *GmDREB1* transgenic wheat.

There have been many studies examining the downstream regulation effects of *DREB*-like genes in Arabidopsis, and it is known that these transcription factors control the expression of stress-related genes including *HsfA3*, *rd29A/cor78*, *kin1*, *kin2*, *cor6.6/kin2*, *cor15a*, *cor47/rd17,* and *erd10*, and result in comprehensive improvements in tolerance to multiple stressors in transgenic plants (Liu *et al.*, 1998; Seki *et al.*, 2001; Maruyama *et al.*, 2004; Zhao *et al.*, 2007). In our present study we found that *GmDREB1* affected plant drought tolerance by regulating melatonin-related signaling pathways. The biomolecular details of melatonin-related signaling pathway(s) are currently not fully understood; hence, our study provides contextual insights that may help to further elucidate melatonin and DREB-related signaling pathways in plants.

### Transgenic expression of *GmDREB1* prolongs leaf functional duration

Senescence is the final stage of leaf development in nature, and is an important determinant of crop yield. Delaying leaf senescence and extending the duration of leaf photosynthesis during grain-filling is a possible route for increasing yields (Hu *et al.*, 2018). Continuous water deficit can induce premature senescence of leaves, and total soluble proteins, Rubisco, and GS isoforms in leaves can be used as indicative traits to determine the onset and stage of senescence as mediated by chloroplast stability (Khanna-Chopra, 2012). Our results showed that under drought stress the total protein content and GS activity of flag leaves in the transgenic plants were higher than the next (second) leaf down the stem (Fig. 4B, D), and we observed that symptoms of senescence first appeared on the older leaves and then on the younger leaves. In contrast, in WT plants the protein content and GS activity were lower in the flag leaf than in the second leaf, indicating that senescence first appeared in the younger leaves in these plants. SDS-PAGE analysis showed that degradation of the rubisco large subunit in the flag leaves of the WT was more obvious than for the transgenic lines (Fig. 4E). The transgenic plants senesced more slowly than the WT, especially in the flag leaves, and the light interception efficiency (LIE) would therefore have been higher than that of WT plants. In addition, the degree of senescence in the transgenic plants after the flowering stage under drought stress was delayed compared with the WT, as evidenced by the higher chlorophyll contents that we observed (Fig. 2F). Therefore, we propose that the increased LIE and leaf area index after the flowering stage that resulted from the delayed leaf senescence were the major determinants underlying the observed improvements in crop biomass and yield in the transgenic plants.

### Conclusions

In summary, through field experiments across a range of years, sites, and drought-stress regimes, we have demonstrated that overexpression of soybean *GmDREB1* can significantly improve the yield of transgenic wheat under drought conditions. We found that *GmDREB1* regulated the synthesis of melatonin in the transgenic plants, and that increased melatonin content appeared to reduce the inhibitory impact of drought stress on the development of the root system. The resulting increased root system was advantageous for increasing water uptake. In addition, the rate of water loss in the transgenic plants was significantly lower than that in the wild-type. The resulting improved water status of the transgenic plants may have contributed to their relatively more stable chloroplasts and delayed senescence compared to the wild-type. A delay in ageing conferred an advantage for increased accumulation of biomass in the transgenic plants under drought conditions, and ultimately increased the grain yield.

## Supplementary data

Supplementary data are available at *JXB* online.

Fig. S1. Illustration of the experimental set-up in greenhouses and rainproof shelters.

Fig. S2. Screening of drought tolerance in the T_2_ generation of the *GmDREB1* transgenic line T349.

Fig. S3. Southern blotting for *GmDREB1* in the T_4_ generation lines.

Fig. S4. Illustration of the field performance of Jimai20 and the corresponding transgenic lines under non-irrigated conditions.

Fig. S5. Physiological parameters showing improved performance of *GmDREB1* transgenic wheat in rainproof shelters under drought conditions.

Fig. S6. Grain yield responses of individual genotypes to the environmental index (mean yield of all cultivars in each environment).

Fig S7. Relationships between grain yield and spikes m^–2^, grains per spike, and thousand-grain weight for the various genotypes across all the sites and treatments.

Fig. S8. Grains m^–2^ under well-irrigated and limited-irrigation conditions across all the sites in 2012.

Fig. S9. Root morphological parameters of the Jimai19 wild-type and the transgenic lines grown hydroponically under normal and stressed conditions.

Fig S10. The root activity of different root layers during the grain-filling stage, as determined by α-Naphthylamine oxidation.

Fig. S11. Rainfall measured in each year during the growing period at the three experimental sites.

Table S1. Regeneration statistics for *GmDREB1*-overexpressing transgenic wheat from the T_0_ and T_1_ generations.

Table S2. Yield results for all the genotypes and drought treatments at the three experimental sites in each of the years of study.

Table S3. Yield components for all the genotypes and drought treatments at the three experimental sites in each of the years of study.

Table S4. Shoot dry weights at different growth stages and grain yield for Jimai19 and transgenic T349 plants in response to drought treatments at the Shijiazhuang site in 2013.

Table S5. Root morphological parameters for Jimai19 and transgenic T349 plants grown in root tubes under well-watered and drought treatments at the Shijiazhuang site in 2013.

Table S6. Root distribution in different soil layers of Jimai19 and transgenic T349 plants at the jointing stage grown in root tubes and subjected to well-watered or drought conditions at the Shijiazhuang site in 2013.

Table S7. Root distribution in different soil layers of Jimai19 and transgenic T349 plants at the flowering stage grown in root tubes and subjected to well-watered or drought conditions at the Shijiazhuang site in 2013.

Table S8. Differentially expressed genes between wild-type Jimai19 and transgenic T349 under well-watered conditions.

Table S9. Differentially expressed genes between wild-type Jimai19 and transgenic T349 under drought conditions.

Table S10. GO enrichment analysis of differentially expressed genes between wild-type Jimai19 and transgenic T349 under well-watered conditions.

Table S11. GO enrichment analysis of differentially expressed genes between wild-type Jimai19 and transgenic T349 under drought conditions.

Table S12. Soil water content during the 2013 growing season at the three experimental sites.

Table S13. Plant height and panicle length for all the genotypes and drought treatments at the three experimental sites in each of the years of study.

Table S14. Main growth periods for the genotypes at the three experimental sites under well-watered conditions in the 2010–2011 and 2011–2012 growing seasons.

Table S15. List of primers used for the stress-responsive genes.

erz569_suppl_supplementary_figures_S1_S11_tables_S1_S7_S12_S15

erz569_suppl_supplementary_table_S8

erz569_suppl_supplementary_table_S9

erz569_suppl_supplementary_table_S10

erz569_suppl_supplementary_table_S11
