# Supplementary material for: Overexpression of soybean DREB1 enhances drought stress tolerance of transgenic wheat in the field
Source: J Exp Bot. 2019 Dec 26;71(6):1842–57. doi: 10.1093/jxb/erz569 (PMC7242075; doi:10.1093/jxb/erz569)
Supplement: erz569_suppl_supplementary_figures_S1_S11_tables_S1_S7_S12_S15 [file erz569_suppl_supplementary_figures_s1_s11_tables_s1_s7_s12_s15.pdf]

Overexpression of the soybean (*Glycine max*) DRE-binding transcription factor *GmDREB1* enhanced drought stress tolerance of transgenic wheat in the field

Yongbin Zhou[1, †], Ming Chen[2, †], Jinkao Guo[3], Yanxia Wang[3], Donghong Min[1], Qiyan Jiang[2], Hutai Ji[4], Chengyan Huang[5], Wei Wei[2], Huijun Xu[2], Xiao Chen[2], Liancheng Li[2], Zhaoshi Xu[2], Xianguo Cheng[2], Chunxiao Wang[2], Chengshe Wang[1, *] and Youzhi Ma[2]

Supplementary materials

Fig. S1 Drought tolerance studies of transgenic wheat conducted in a greenhouse and using a rainproof shelter.

Fig. S2 Drought tolerance screening of T2 generation *GmDREB1* transgenic lines T349 (TG).

Fig. S3 Southern blotting for *GmDREB1* gene in T4 generation lines using two restriction enzymes, *BamH*I and *Hind*III, to cut the genomic DNA.

Fig. S4 Field performance of transgenic lines (CM7 and CM14) and the wild type (Jm20) during the 2012 wheat-growing season in Shijiazhuang under non-irrigated condition (NIR).

Fig. S5 *GmDREB1* transgenic wheat exhibited improvements in a variety of physiological traits in rainproof shelter.

Fig. S6 Grain yield response of individual genotypes to the environmental index.

Fig S7 Grain yield response to its components, namely spikes/m^2, grains/spike, and 1000-grains weight (TGW).

Fig S8 Grains/m^2 in all environments.

Fig. S9 Root morphological parameters of the wild type (Jm19) and the transgenic lines (T349 and T398) grown hydroponically (normal and drought).

Fig S10 The root activity of different root layers was measured during grain filling stage

Fig. S11 Rainfall during growing period.

Table S1 Regeneration of *Ubi::GmDREB1*-Overexpressing transgenic wheat from T0-T1 in Jimai19 (Jm19) and Jimai20 (Jm20).

Table S2 Yield results of transgenic wheat lines and WT in three locations including Shijiazhuang

city, Jinan city, and Linfen city from 2011 to 2013.

Table S3 Yield components of transgenic wheat lines and WT in the field.

Table S4 Shoot dry weight at jointing and flowering, and at maturity, and grain yield (GY) for TG (T349) and WT (Jm19) plants exposed to drought stress

Table S5 Root morphological parameters of the WT (Jm19) and the TG (T349) grown in root tube (0-240cm) under normal and drought treatment, respectively.

Table S6 Root distribution of TG (T349) and WT in different root layers during jointing stage.

Table S7 Root distribution of TG (T349) and WT in different root layers during flowering stage.

Table S8 T349-vs-WT-diffierent gene (normal treatment).

Table S9 T349-vs-WT-diffierent gene (drought treatment).

Table S10 GO-enrichment-T349-vs-WT(normal treatment).

Table S11 GO-enrichment-T349-vs-WT(drought treatment).

Table S12 Soil water content during the growing season (2013).

Table S13 Variety of plant height (PH) and panicle length (PL) under different irrigation conditions in the field.

Table S14 Main growth period of T4 generation transgenic wheat lines and WT under WIR in the 2012 growing season.

Table S15 All primers for the stress-responsive genes.

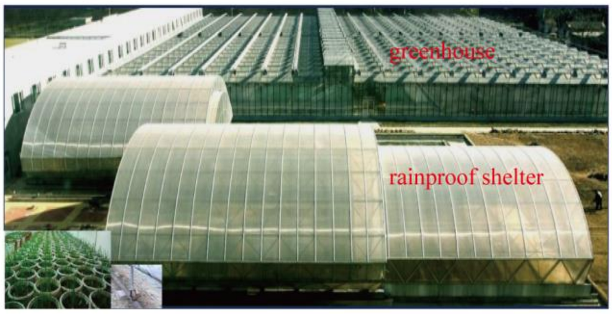

Fig. S1 Drought tolerance studies of transgenic wheat conducted in a greenhouse and using a rainproof shelter.

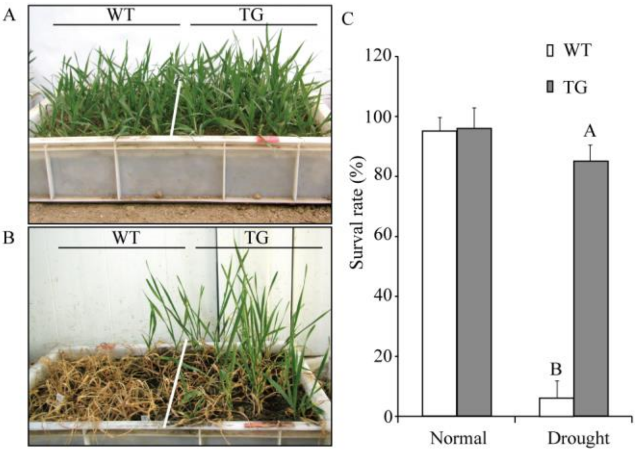

Fig. S2 Drought tolerance screening of T2 generation *GmDREB1* transgenic lines T349 (TG). (A) Phenotypes of transgenic wheat grown under normal conditions for three weeks. (B) Phenotypes of the transgenic wheat under drought for 45 days, after rehydration for 20 days. (C) Survival rates of the transgenic lines T349 plants and WT plants at P < 0.01.

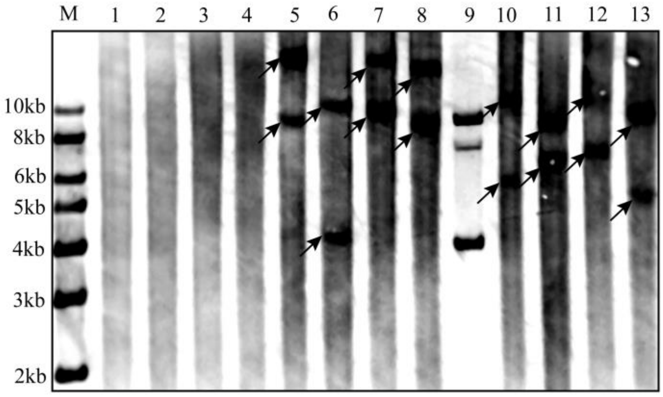

Fig. S3 Southern blotting for *GmDREB1* gene in T4 generation lines using two restriction enzymes, *BamH*I and *Hind*Ⅲ, to cut the genomic DNA. M, marker; 1 and 2, negative control (Jimai19); 3 and 4, negative control (Jimai20); 5 and 10, T349 line; 6 and 11, T398 line; 7 and 12, CM7 line; 8 and 13, CM14 line; 9, positive control (plasmid); 1, 3, 5, 6, 7 and 8, using *BamH*I restriction enzyme; 2, 4, 10, 11, 12 and 13 using *Hind*Ⅲ restriction enzyme.

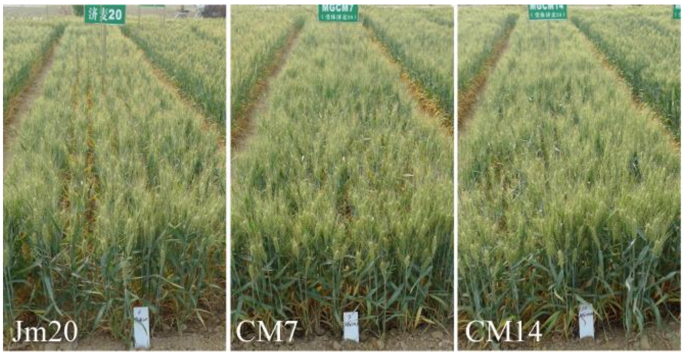

Fig. S4 Field performance of transgenic lines (CM7 and CM14) and the wild type (Jm20) during the 2012 wheat-growing season in Shijiazhuang under non-irrigated condition (NIR).

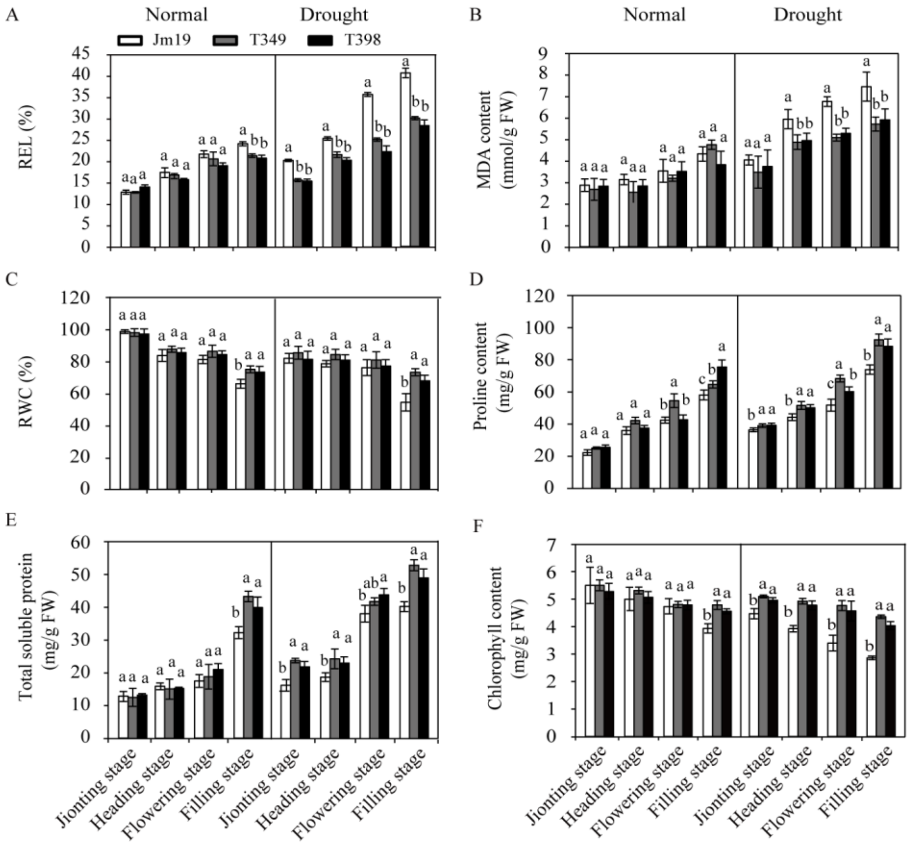

Fig. S5 *GmDREB1* transgenic wheat exhibited improvements in a variety of physiological traits in rainproof shelter. The germinated seedlings were grown in plastic pots (25×45 cm) filled with surface soil and pot were placed in rainproof shelter. Rainproof shelters were typically opened, except during rain. Experiments were divided into two groups: a drought treatment group for which all of the transgenic lines and WT experienced drought stress (the relative soil water content of 40-45%) at the jointing stage, heading stage, flowering stage, and grain filling stage; and the normal treatment group, for which all plants experienced adequate water intake (relative soil water content of 80-85%) at the jointing stage, heading stage, flowering stage, and grain filling stage. All indexes for transgenic wheat and WT were determined in flag leaves following soil water measurements. (A) Relative electrolyte leakage (REL) in flag leaves under normal and

drought treatment. (B) MDA content in flag leaves under normal and drought treatment. (C) Relative water content (RWC) in flag leaves under normal and drought treatment. (D) Proline content in flag leaves under normal and drought treatment. (E) Total soluble protein in flag leaves under normal and drought treatment. (F) Chlorophyll content in flag leaves under normal and drought treatment. Data represent means ± SE of three replicates. Different letters indicate that the difference between the means of the transgenic lines and wild type was significant at the $P < 0.05$ (a, b, and c) and $P < 0.01$ (A, B, and C) level.

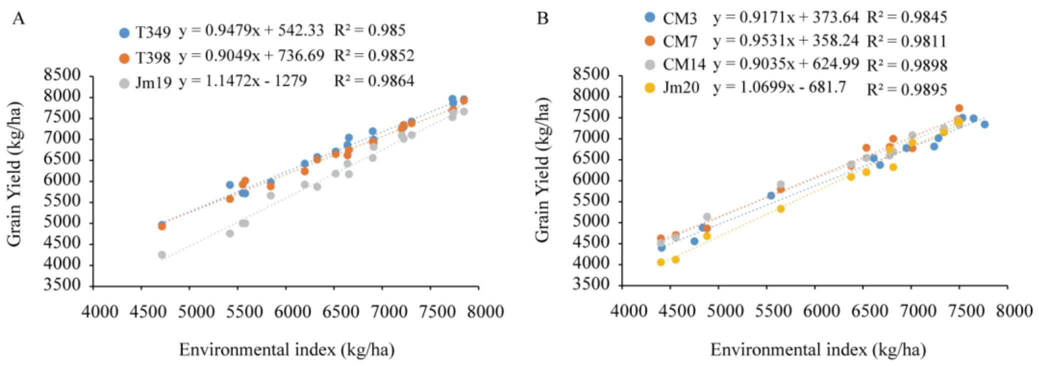

Fig. S6 Grain yield response of individual genotypes to the environmental index. (A) The regression coefficient of yield of Jm19 and transgenic lines (T349 and T398) at the three tested sites. (B) The regression coefficient of yield of Jm20 and transgenic lines (CM3, CM7, and CM14)

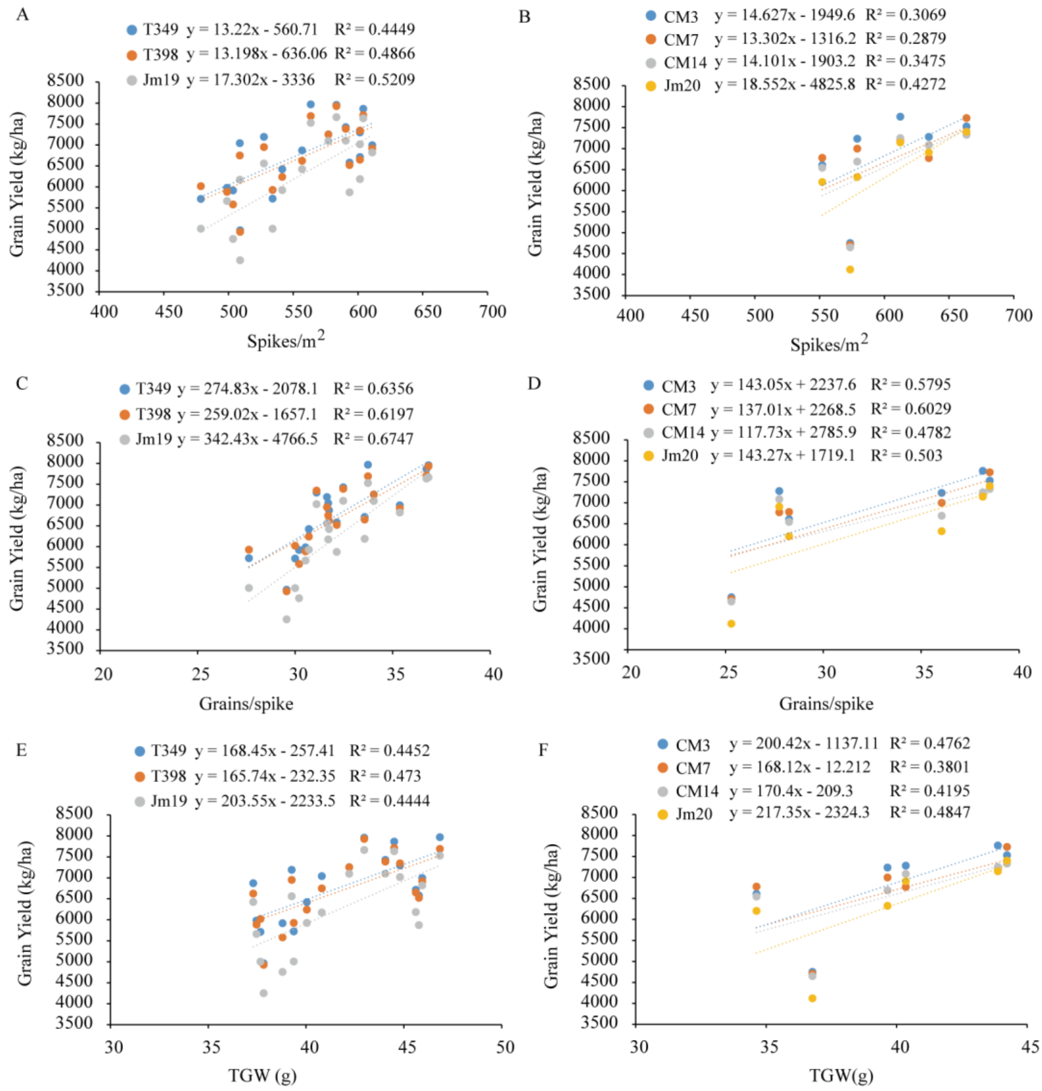

Fig S7 Grain yield response to its physiological determinants, namely spikes/m^2, grains/spike, and 1000-grains weight (TGW). (A) – (B) The regression coefficient of yield of and spikes/m^2. (C) - (D) The regression coefficient of yield of and grains/spike. (E) – (F) The regression coefficient of yield of and TGW.

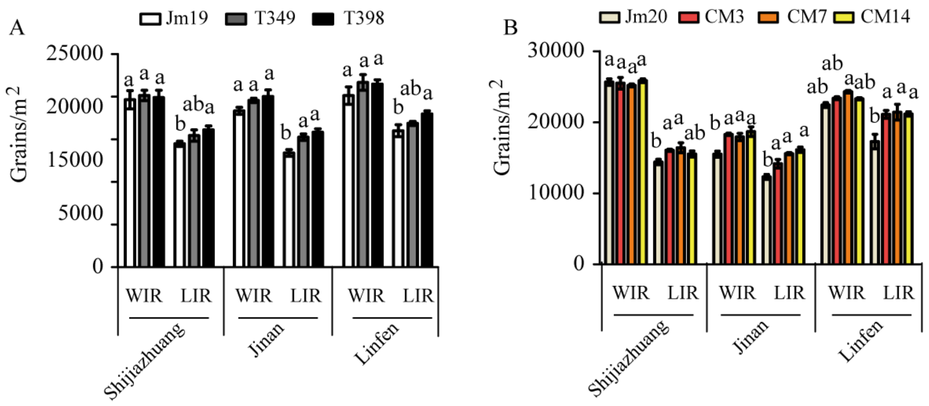

Fig S8 Grains/m^2 in all environments. (A) Grains/m^2 in three locations under group (Jm19+TGs). (B) Grains/m^2 in three locations under group (Jm20+TGs). Different letters indicate that the difference between the means of the transgenic lines and wild type was significant at the P < 0.05 (a, b and c) level.

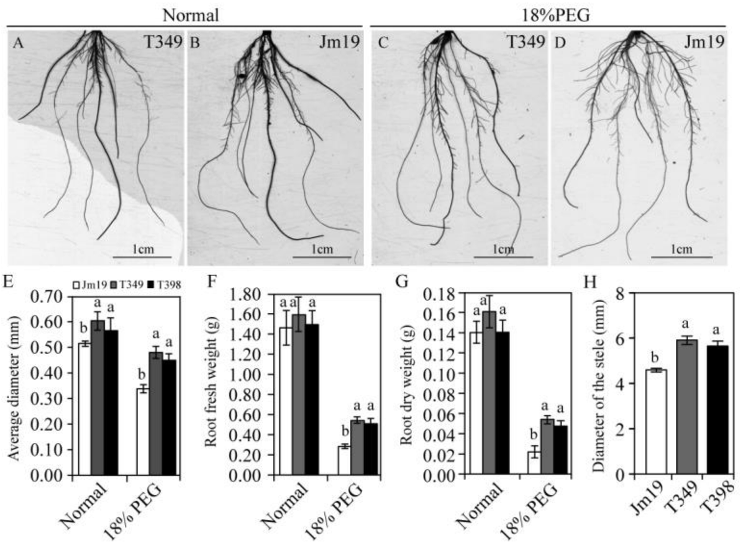

Fig. S9 Root morphological parameters of the wild type (Jm19) and the transgenic lines (T349 and T398) grown hydroponically (normal and drought). Wheat seedlings were grown for 14 d in the normal nutrient solution and 18% PEG-6000 nutrient solution, and then the roots were scanned with an STD1600 scanner and analyzed for the root morphological parameters with WinRHIZO software (Regent Instruments). (A)—(D) Root images of the wild type and T349. (E) Average of root diameter in root scanner (mm). (F) Root fresh weight (g). (G) Root dry weight (g). (H) Diameter of the stele. Data represent means ± SE of three replicates. Different letters indicate that the difference between the means of the transgenic lines and wild type was significant at the P < 0.05 (a, b and c) level.

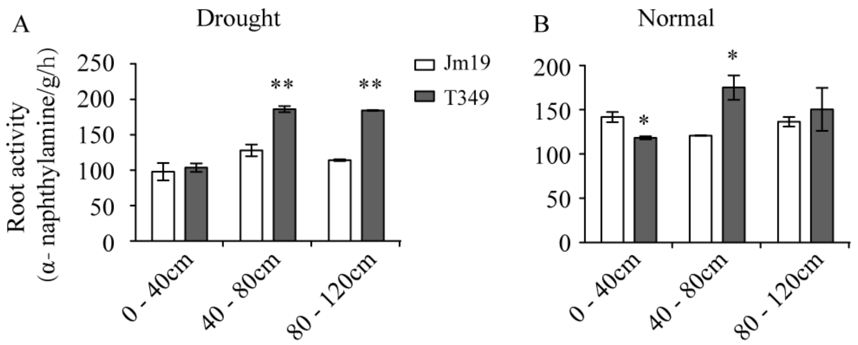

Fig S10. The root activity of different root layers was measured during grain filling stage. (A) The root activity of different root layers in drought treatment. (B) The root activity of different root layers in normal treatment. * and ** indicate that difference between the WT and the TG was significant at the $P < 0.05$ and 0.01 level

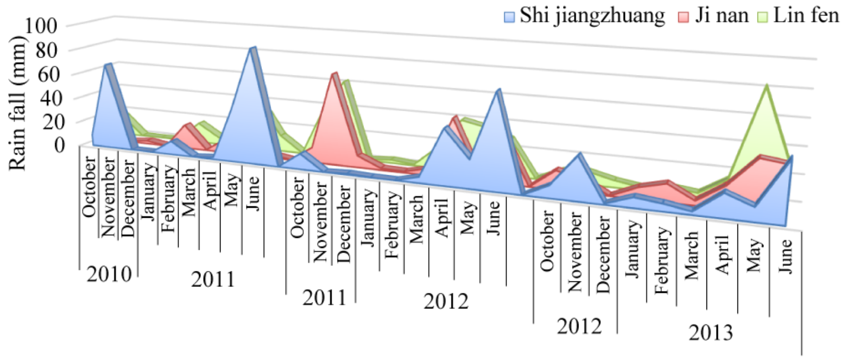

Fig. S11 Rainfall during growing period. The receptor is winter wheat, the whole growth period is from October to June of next year.

Table S1 Regeneration of *Ubi::GmDREB1*-Overexpressing transgenic wheat from T0-T1 in Jimai19 (Jm19) and Jimai20 (Jm20)

receptor	T0						T1		
	callus	Regenerated plants	fertile plants	PCR positive plants	Transformation frequency	PCR positive ratios	Plants	PCR positive plants	PCR positive ratios
Jm19	1715	170	131	51	3.0%	30.0%	186	74	39.8%
Jm20	490	14	10	4	0.8%	28.6%	82	41	50%

Table S2 Yield results of transgenic wheat lines and WT in three locations including Shijiazhuang city, Jinan city, and Linfen city from 2011 to 2013.

Lines	Shijiazhuang														
	2010—2011		2011—2012			2012—2013			Mean			YI (%)		
	LIR	WIR	NIR	LIR	WIR	NIR	LIR	WIR	NIR	LIR	WIR	NIR	LIR	WIR
T349	5982.30±64.23^{Aa}	6995.57±56.67^{a}	4913.83±126.48^{a}	7043.56±133.37^{Aa}	7969.53±230.46^{a}	nd	5723.05±52.31^{A}	7426.86±45.57^{a}	4913.83±126.48^{a}	6249.64±207.00^{a}	7463.99±157.21^{a}	12.51	11.31	4.38
T398	5883.97±49.36^{Ba}	6917.72±82.80^{a}	4802.71±37.55^{a}	6750.34±126.40^{ABa}	7691.74±145.17^{a}	nd	5927.72±100.89^{A}	7387.07±167.49^{a}	4802.71±37.55^{a}	6187.34±149.11^{a}	7332.18±131.66^{a}	9.96	10.20	2.53
Jm19	5665.61±49.28^{Bb}	6821.95±58.28^{a}	4367.50±163.94^{b}	6173.15±72.58^{Bb}	7528.15±137.03^{a}	nd	5005.20±88.33^{B}	7102.78±212.90^{a}	4367.50±163.94^{b}	5614.65±172.84^{b}	7150.96±127.12^{a}			
CM3	nd	nd	5018.80±92.49^{a}	6610.05±109.75^{ABa}	7531.30±123.96^{a}	4835.79±236.05^{a}	5547.35±85.35^{ABab}	7165.28±161.18^{a}	4927.29±120.54^{a}	6078.70±245.63^{a}	7348.29±122.34^{a}	4.23	5.40	0.59
CM7	nd	nd	4826.48±37.74^{a}	6783.75±127.02^{Aa}	7729.81±145.89^{a}	4866.81±153.19^{a}	5795.81±26.37^{Bb}	7469.26±24.81^{a}	4846.64±71.13^{a}	6289.78±228.40^{a}	7599.54±88.17^{a}	2.53	9.06	4.03
CM14	nd	nd	4984.68±123.72^{a}	6544.91±45.06^{ABa}	7329.68±79.75^{a}	5144.42±85.64^{a}	5918.33±105.83^{Bb}	7314.17±130.28^{a}	5064.55±76.19^{a}	6231.62±149.25^{a}	7321.92±68.40^{a}	7.14	8.05	0.23
Jm20	nd	nd	4773.75±153.19^{a}	6203.70±58.94^{Bb}	7404.12±236.29^{a}	4680.69±84.44^{a}	5330.53±119.42^{Aa}	7205.60±67.17^{a}	4727.22±80.95^{a}	5767.12±204.13^{b}	7304.86±118.49^{a}			

Lines	Jinan									
	2010—2011		2011—2012		2012—2013		Mean		YI (%)	
	LIR	WIR	LIR	WIR	LIR	WIR	LIR	WIR	LIR	WIR
T349	4965.80±126.48^{a}	7299.01±57.23^{a}	5713.40±81.93^{a}	6581.15±191.81^{a}	5918.50±74.16^{A}	6715.60±164.84^{a}	5532.57±152.66^{A}	6865.25±133.19^{a}	18.43	7.95
T398	4927.68±80.64^{a}	7346.22±160.88^{a}	6020.03±238.61^{a}	6523.37±140.49^{a}	5581.50±108.59^{A}	6651.15±117.40^{ab}	5509.74±177.34^{A}	6840.24±145.91^{a}	17.94	7.56
Jm19	4250.83±180.46^{b}	7019.89±353.08^{a}	5004.45±249.01^{b}	5872.30±211.10^{b}	4760.00±131.15^{B}	6186.80±121.02^{b}	4671.76±147.08^{B}	6359.66±211.25^{a}		
CM3	nd	nd	4750.30±103.20^{a}	7283.29±177.48^{a}	4410.00±36.37^{ABa}	6951.00±145.82^{a}	4580.15±90.47 ^{a}	7117.15±126.78 ^{a}	12.00	4.22

CM7	nd	nd	4706.75±177.27^{a}	6774.88±354.18^{a}	4629.00±153.81^{Aa}	6805.50±36.57^{a}	4667.88±106.39 ^{a}	6790.19±159.38 ^{a}	14.14	-0.56
CM14	nd	nd	4649.80±159.57^{a}	7086.24±208.44^{a}	4514.00±63.62^{ABa}	6600.00±122.31^{a}	4581.90±82.61 ^{a}	6843.12±153.30 ^{a}	12.04	0.21
Jm20	nd	nd	4120.50±58.02^{b}	6908.88±173.55^{a}	4058.50±100.56^{Bb}	6748.50±144.30^{a}	4089.50±53.74 ^{b}	6828.69±107.12 ^{a}		
	Linfen									
Lines	2010—2011		2011—2012		2012—2013		Mean		YI (%)	
	LIR	WIR	LIR	WIR	LIR	WIR	LIR	WIR	LIR	WIR
T349	7193.80±83.49^{Aa}	7865.45±505.85^{a}	6423.70±96.85^{Aa}	7956.30±125.58^{a}	6873.00±64.09^{A}	7256.00±190.80^{a}	6830.17±119.06^{A}	7692.58±194.31^{a}	8.36	3.03
T398	6950.50±96.89^{ABa}	7713.00±137.75^{a}	6240.80±56.16^{ABa}	7928.90±179.03^{a}	6624.90±68.27^{AB}	7253.00±83.11^{a}	6605.40±109.32^{B}	7631.63±121.49^{a}	4.79	2.21
Jm19	6560.45±90.09^{Bb}	7636.95±304.48^{a}	5925.45±44.90^{Bb}	7663.55±129.24^{a}	6424.50±55.43^{B}	7098.30±339.50^{a}	6303.47±102.06^{B}	7466.27±164.92^{a}		
CM3	nd	nd	7238.70±99.55^{Aa}	7763.70±111.37^{a}	6676.65±52.87^{ACa}	7648.50±103.79^{a}	6957.68±135.41 ^{a}	7706.10±72.79 ^{a}	12.10	5.98
CM7	nd	nd	7000.75±77.10^{ABab}	7194.40±284.72^{a}	6352.50±37.54^{Bb}	7456.50±19.11^{ab}	6676.63±149.94 ^{a}	7325.45±140.43^{a}	7.57	0.74
CM14	nd	nd	6691.40±126.91^{BCb}	7252.20±473.55^{a}	6390.00±110.99^{BCb}	7438.50±24.25^{ab}	6540.70±101.13 ^{ab}	7345.35±216.11^{a}	5.38	1.02
Jm20	nd	nd	6323.80±98.54^{Cc}	7147.85±435.25^{a}	6090.00±3.46^{Bc}	7395.00±19.05^{b}	6206.90±68.39 ^{b}	7271.43±202.52^{a}		

The yield (KG per hectare, kg/ha) comparing experiments were completed in treatment of non-irrigated (NIR), limited-irrigated (LIR) and well-irrigated (WIR) in Shijiazhuang (Hebei province), Jinan (Shandong province) and Lin fen (Shan xi province) by three times repeats in 2011–2013 growing season; yield-test results calculated as yield of grains in plot (6.7m^2); YI, yield increased than WT. Data represent means ± SE of three replicates. Different letters indicate that the difference between the means of the transgenic lines and wild type was significant at the $P < 0.05$ (a, b and c) and $P < 0.01$ (A, B and C) level.

Table S3 Yield components of transgenic wheat lines and WT in the field

Treatment	Lines	Shijiazhuang				Jinan				Linfen			
		SN											
		2010—2011	2011—2012	2012—2013	Mean value	2010—2011	2011—2012	2012—2013	Mean value	2010—2011	2011—2012	2012—2013	Mean value
LIR	T349	543.5±9.9^{a}	545.7±16.7^{a}	560.5±7.6^{a}	553.2±6.4^{a}	549.5±14.8^{a}	517.0±8.3^{a}	558.0±15.1^{a}	541.5±9.1^{a}	582.5±13.9^{a}	572.5±13.0^{a}	577.5±9.2^{a}	577.5±6.3^{a}
	T398	517.0±6.6^{a}	527.3±48.0^{a}	541.0±4.4^{a}	528.4±14.5^{a}	518.5±14.9^{a}	501.0±10.5^{a}	510.3±16.2^{b}	509.9±7.5^{a}	518.1±9.2^{b}	568.5±9.8^{a}	570.0±3.0^{a}	552.2±9.4^{b}

	**Jm19**	**436.0±16.8^{b}**	**453.1±11.0^{b}**	**500.5±9.5^{b}**	**463.2±11.6^{b}**	**459.0±6.9^{b}**	**418.0±8.8^{b}**	**442.0±8.1^{c}**	**439.6±7.2^{b}**	**481.5±14.3^{b}**	**483.5±16.1^{b}**	**523.0±7.2^{b}**	**496.0±9.4^{c}**
	**CM3**	**nd**	**554.0±5.9^{a}**	**nd**	**554.0±5.9^{a}**	**nd**	**558.0±23.1^{ab}**	**nd**	**558.0±23.1^{ab}**	**nd**	**565.5±0.0^{ab}**	**nd**	**565.5±0.0^{ab}**
	**CM7**	**nd**	**565.5±3.1^{a}**	**nd**	**565.5±3.1^{a}**	**nd**	**599.0±3.6^{a}**	**nd**	**599.0±3.6^{a}**	**nd**	**594.2±14.2^{a}**	**nd**	**594.2±14.2^{a}**
	**CM14**	**nd**	**555.0±2.3^{a}**	**nd**	**555.0±2.3^{a}**	**nd**	**608.0±19.5^{a}**	**nd**	**608.0±19.5^{a}**	**nd**	**577.5±10.4^{ab}**	**nd**	**577.5±10.4^{ab}**
	**Jm20**	**nd**	**534.0±3.8^{b}**	**nd**	**534.0±3.8^{b}**	**nd**	**529.3±6.12^{b}**	**nd**	**529.3±6.12^{b}**	**nd**	**505.8±30.5^{b}**	**nd**	**505.8±30.5^{b}**
**WIR**	**T349**	**623.8±11.6^{a}**	**602.5±19.0^{a}**	**624.0±16.0^{a}**	**616.8±8.7^{a}**	**642.1±13.3^{a}**	**612.0±12.5^{a}**	**623.1±5.5^{a}**	**625.7±7.3^{a}**	**630.0±22.7^{a}**	**607.5±15.9^{a}**	**596.8±11.0^{a}**	**611.4±10.0^{a}**
	**T398**	**613.9±13.6^{a}**	**562.7±14.1^{ab}**	**575.0±14.6^{b}**	**583.8±10.5^{ab}**	**607.1±10.3^{a}**	**604.0±17.6^{ab}**	**615.6±14.8^{a}**	**608.9±7.5^{a}**	**596.3±13.7^{a}**	**601.7±17.6^{ab}**	**593.5±10.8^{ab}**	**597.2±7.2^{a}**
	**Jm19**	**595.4±13.2^{a}**	**525.2±7.8^{b}**	**572.5±11.0^{b}**	**564.4±11.7^{b}**	**555.3±11.3^{b}**	**564.3±6.2^{b}**	**565.9±17.3^{b}**	**561.9±6.4^{b}**	**585.8±4.4^{a}**	**540.8±19.5^{b}**	**541.0±16.4^{b}**	**555.9±10.6^{b}**
	**CM3**	**nd**	**665.0±7.9^{a}**	**nd**	**665.0±7.9^{a}**	**nd**	**642.0±15.0^{a}**	**nd**	**642.0±15.0^{a}**	**nd**	**616.0±12.6^{a}**	**nd**	**616.0±12.6^{a}**
	**CM7**	**nd**	**663.0±2.3^{a}**	**nd**	**663.0±2.3^{a}**	**nd**	**649.0±17.2^{a}**	**nd**	**649.0±17.2^{a}**	**nd**	**634.5±10.0^{a}**	**nd**	**634.5±10.0^{a}**
	**CM14**	**nd**	**663.0±1.5^{a}**	**nd**	**663.0±1.5^{a}**	**nd**	**636.0±17.3^{a}**	**nd**	**636.0±17.3^{a}**	**nd**	**604.1±9.3^{a}**	**nd**	**604.1±9.3^{a}**
	**Jm20**	**nd**	**663.0±2.6^{a}**	**nd**	**663.0±2.6^{a}**	**nd**	**610.3±9.5^{a}**	**nd**	**610.3±9.5^{a}**	**nd**	**594.7±11.3^{a}**	**nd**	**594.7±11.3^{a}**
**Treatment**	**Lines**	**NGPS**											
**LIR**	**T349**	**29.1±1.4^{a}**	**29.5±1.1^{a}**	**25.2±0.2^{a}**	**27.9±0.9^{a}**	**28.9±0.3^{a}**	**28.2±0.8^{a}**	**27.4±0.7^{a}**	**28.2±1.1^{a}**	**29.6±1.2^{a}**	**28.7±0.7^{a}**	**29.3±1.4^{a}**	**29.2±0.6^{a}**
	**T398**	**30.2±0.7^{a}**	**32.3±1.1^{a}**	**29.0±1.1^{a}**	**30.5±0.7^{b}**	**29.3±1.5^{a}**	**31.5±0.8^{b}**	**32.5±0.4^{b}**	**31.1±1.9^{b}**	**33.5±0.8^{a}**	**31.7±0.7^{b}**	**32.6±0.8^{a}**	**32.6±0.5^{b}**
	**Jm19**	**32.3±0.6^{a}**	**33.3±0.9^{a}**	**28.7±1.6^{a}**	**31.4±0.9^{b}**	**30.5±1.2^{a}**	**30.3±0.5^{ab}**	**30.7±0.6^{b}**	**30.5±1.1^{b}**	**31.8±0.7^{a}**	**31.7±0.7^{b}**	**33.3±0.8^{a}**	**32.3±0.4^{b}**
	**CM3**	**nd**	**29.0±0.5^{a}**	**nd**	**29.0±0.5^{a}**	**nd**	**25.4±0.5^{a}**	**nd**	**25.4±0.5^{a}**	**nd**	**37.3±1.0^{a}**	**nd**	**37.3±1.0^{a}**
	**CM7**	**nd**	**29.0±1.1^{a}**	**nd**	**29.0±1.1^{a}**	**nd**	**26.0±0.2^{a}**	**nd**	**26.0±0.2^{a}**	**nd**	**36.0±1.0^{a}**	**nd**	**36.0±1.0^{a}**
	**CM14**	**nd**	**28.0±0.7^{a}**	**nd**	**28.0±0.7^{a}**	**nd**	**26.6±0.8^{a}**	**nd**	**26.6±0.8^{a}**	**nd**	**36.7±0.4^{a}**	**nd**	**36.7±0.4^{a}**
	**Jm20**	**nd**	**27.0±0.8^{a}**	**nd**	**27.0±0.8^{a}**	**nd**	**23.2±0.4^{b}**	**nd**	**23.2±0.4^{b}**	**nd**	**34.2±0.6^{a}**	**nd**	**34.2±0.6^{a}**
**WIR**	**T349**	**34.3±1.0^{a}**	**32.6±1.3^{a}**	**31.2±0.6^{a}**	**32.7±0.7^{a}**	**30.4±1.1^{a}**	**31.3±1.1^{a}**	**32.1±0.8^{a}**	**31.3±1.6^{a}**	**36.4±0.4^{a}**	**36.5±0.6^{a}**	**33.3±1.0^{a}**	**35.4±0.6^{a}**
	**T398**	**35.2±0.9^{a}**	**33.8±3.6^{a}**	**33.2±0.6^{a}**	**34.1±1.1^{a}**	**31.2±0.2^{a}**	**32.5±0.7^{a}**	**35.0±0.7^{a}**	**32.9±1.8^{a}**	**36.3±0.8^{a}**	**37.0±0.9^{a}**	**34.7±1.0^{a}**	**36.0±0.6^{a}**
	**Jm19**	**36.6±1.8^{a}**	**34.8±0.8^{a}**	**33.0±0.6^{a}**	**34.8±0.8^{a}**	**31.7±0.3^{a}**	**32.6±0.5^{a}**	**33.6±0.5^{a}**	**32.7±1.0^{a}**	**37.5±0.8^{a}**	**37.0±0.8^{a}**	**34.1±0.3^{a}**	**36.2±0.6^{a}**
	**CM3**	**nd**	**38.3±0.8^{a}**	**nd**	**38.3±0.8^{a}**	**nd**	**28.5±0.7^{a}**	**nd**	**28.5±0.7^{a}**	**nd**	**38.0±1.0^{a}**	**nd**	**38.0±1.0^{a}**
	**CM7**	**nd**	**38.0±0.4^{a}**	**nd**	**38.0±0.4^{a}**	**nd**	**27.7±1.0^{ab}**	**nd**	**27.7±1.0^{ab}**	**nd**	**38.3±0.7^{a}**	**nd**	**38.3±0.7^{a}**

	**CM14**	**nd**	**39.0±0.3^{a}**	**nd**	**39.0±0.3^{a}**	**nd**	**29.4±1.1^{a}**	**nd**	**29.4±1.1^{a}**	**nd**	**38.6±0.6^{a}**	**nd**	**38.6±0.6^{a}**
	**Jm20**	**nd**	**38.7±0.6^{a}**	**nd**	**38.7±0.6^{a}**	**nd**	**25.4±0.8^{b}**	**nd**	**25.4±0.8^{b}**	**nd**	**37.7±0.5^{a}**	**nd**	**37.7±0.5^{a}**
**Treatment**	**Lines**	**TGW**											
**LIR**	**T349**	**40.3±0.8^{a}**	**42.7±0.3^{a}**	**41.4±0.6^{a}**	**41.5±0.4^{a}**	**39.1±0.3^{a}**	**39.9±0.5^{a}**	**40.6±0.6^{a}**	**39.8±0.9^{a}**	**41.3±0.6^{a}**	**41.5±0.5^{a}**	**39.2±0.3^{a}**	**40.6±0.4^{a}**
	**T398**	**37.1±0.3^{b}**	**42.0±0.8^{a}**	**40.4±0.5^{a}**	**39.9±0.8^{a}**	**37.9±1.3^{a}**	**37.8±0.2^{b}**	**40.9±0.8^{a}**	**38.9±1.9^{a}**	**39.2±0.8^{ab}**	**40.6±0.8^{a}**	**37.8±1.0^{a}**	**39.2±0.6^{b}**
	**Jm19**	**35.0±0.1^{c}**	**37.7±1.0^{b}**	**36.3±1.3^{b}**	**36.3±0.6^{b}**	**36.5±0.8^{ab}**	**35.3±0.6^{c}**	**34.9±0.7^{b}**	**35.6±1.2^{b}**	**37.3±1.2^{b}**	**38.0±0.5^{b}**	**34.9±0.1^{b}**	**36.7±0.6^{c}**
	**CM3**	**nd**	**35.7±0.2^{a}**	**nd**	**35.7±0.2^{a}**	**nd**	**36.9±0.8^{a}**	**nd**	**36.9±0.8^{a}**	**nd**	**40.2±0.9^{a}**	**nd**	**40.2±0.9^{a}**
	**CM7**	**nd**	**34.6±0.3^{ab}**	**nd**	**34.6±0.3^{ab}**	**nd**	**37.7±0.7^{a}**	**nd**	**37.7±0.7^{a}**	**nd**	**39.7±0.7^{a}**	**nd**	**39.7±0.7^{a}**
	**CM14**	**nd**	**35.7±0.2^{a}**	**nd**	**35.7±0.2^{a}**	**nd**	**36.7±0.3^{a}**	**nd**	**36.7±0.3^{a}**	**nd**	**41.3±2.1^{a}**	**nd**	**41.3±2.1^{a}**
	**Jm20**	**nd**	**32.5±0.9^{b}**	**nd**	**32.5±0.9^{b}**	**nd**	**35.8±0.6^{a}**	**nd**	**35.8±0.6^{a}**	**nd**	**37.4±0.7^{a}**	**nd**	**37.4±0.7^{a}**
**WIR**	**T349**	**47.1±0.5^{a}**	**47.6±0.9^{a}**	**45.8±1.5^{a}**	**46.8±0.6^{a}**	**45.7±0.6^{a}**	**46.5±0.9^{a}**	**46.5±1.0^{a}**	**46.2±1.3^{a}**	**44.5±0.8^{a}**	**43.9±0.5^{a}**	**42.5±0.8^{a}**	**43.6±0.5^{a}**
	**T398**	**44.9±0.9^{a}**	**47.0±0.9^{a}**	**44. 4±1.2^{a}**	**45.4±0.7^{a}**	**44.2±0.5^{a}**	**45.2±0.7^{a}**	**45.2±0.6^{a}**	**44.9±1.0^{a}**	**44.7±0.7^{a}**	**42.7±0.4^{a}**	**42.7±0.5^{a}**	**43.4±0.4^{a}**
	**Jm19**	**45.8±0.6^{a}**	**45.9±0.8^{a}**	**42.3±0.9^{a}**	**44.7±0.7^{a}**	**44.5±1.0^{a}**	**45.6±0.5^{a}**	**45.1±0.7^{a}**	**45.1±1.2^{a}**	**44.3±1.2^{a}**	**42.3±0.8^{a}**	**41.4±0.9^{a}**	**42.7±0.6^{a}**
	**CM3**	**nd**	**43.7±0.3^{a}**	**nd**	**43.7±0.3^{a}**	**nd**	**41.3±1.0^{a}**	**nd**	**41.3±1.0^{a}**	**nd**	**43.3±0.9^{a}**	**nd**	**43.3±0.9^{a}**
	**CM7**	**nd**	**43.4±0.2^{a}**	**nd**	**43.4±0.2^{a}**	**nd**	**40.8±1.1^{a}**	**nd**	**40.8±1.1^{a}**	**nd**	**44.7±0.9^{a}**	**nd**	**44.7±0.9^{a}**
	**CM14**	**nd**	**45.8±0.2^{b}**	**nd**	**45.8±0.2^{b}**	**nd**	**40.0±0.9^{a}**	**nd**	**40.0±0.9^{a}**	**nd**	**44.3±0.8^{a}**	**nd**	**44.3±0.8^{a}**
	**Jm20**	**nd**	**44.0±0.4^{ac}**	**nd**	**44.0±0.4^{ac}**	**nd**	**39.3±0.8^{a}**	**nd**	**39.3±0.8^{a}**	**nd**	**43.2±0.8^{a}**	**nd**	**43.2±0.8^{a}**

TN, the spike number (1 m^2); NGPS, number of grains per spike; TGW, 1000-grains weight. nd, not data. Data represent means ± SE of three replicates. Different letters indicate that the difference between the means of the transgenic lines and wild type was significant at the $P < 0.05$ (a, b and c) level.

Table S4 Shoot dry weight at jointing and flowering, and at maturity, and grain yield (GY) for TG (T349) and WT (Jm19) plants exposed to drought stress

**Treatment**	**Lines**	**Shoot dry weight （g/plant）**			**Δ[F-J] (g/plant)**	**Δ[M-F] (g/plant)**	**GY (g/plant)**	**TGW (g)**	**HI**
		**Jointing**	**Flowering**	**Mature**					
**LIR**	**TG**	**4.61^{a}**	**8.96^{a}**	**44.44^{a}**	**4.35^{a}**	**35.48^{a}**	**18.00^{a}**	**37.00^{a}**	**0.41^{a}**
	**WT**	**3.95^{a}**	**8.77^{b}**	**37.82^{b}**	**4.82^{a}**	**29.05^{b}**	**16.05^{b}**	**34.00^{b}**	**0.42^{a}**

WIR	TG	4.54^{a}	9.54^{a}	51.20^{a}	5.00^{a}	41.67^{a}	22.15^{a}	42.50^{a}	0.43^{a}
	WT	4.61^{a}	9.33^{a}	45.72^{a}	4.72^{a}	36.39^{a}	20.30^{a}	40.50^{a}	0.44^{a}

Shown are the jointing–flowering biomass increase (Δ[F-J]), Post-flowering biomass increase (Δ[M-F]), grain yield per plant (GY), thousand grain weight (TGW), and harvest index (HI) also shown. Within each column, values followed by different letters are significantly ($p \leqslant 0.05$) different

Table S5 Root morphological parameters of the WT (Jm19) and the TG (T349) grown in root tube (0-240cm) under normal and drought treatment, respectively.

Treatment	Lines	Jointing stage			Flowering stage		
		root surface area (mm^2)	Root length (mm)	Root dry weight (g)	root surface area (mm^2)	Root length (mm)	Root dry weight (g)
Drought	TG	36347.47^{a}	60697.06^{a}	0.63^{a}	47743.68^{a}	82356.11^{A}	0.84^{A}
	WT	36216.62^{a}	55645.97^{a}	0.56^{a}	39188.60^{b}	66105.34^{B}	0.57^{B}
Normal	TG	45374.18^{a}	76482.02^{a}	0.68^{a}	58589.26^{a}	101732.25^{a}	0.85^{a}
	WT	43180.69^{a}	75525.85^{a}	0.66^{a}	54992.44^{a}	83065.59^{b}	0.76^{a}

All parameters come from the summation of different root layers (0-240cm). Data represent means ± SE of three replicates. Different letters indicate that the difference between the means of the transgenic lines and wild type was significant at the $P < 0.05$ (a, b and c) and $P < 0.01$ (A, B and C) level.

Table S6 Root distribution of TG (T349) and WT in different root layers during jointing stage

Lines	Soil distribution (cm)	Drought			Normal		
		root surface area (mm^2)	Root length (mm)	Root dry weight (g)	root surface area (mm^2)	Root length (mm)	Root dry weight (g)
TG	0-40	21719.7	35462.2	0.34	29885.43	46647.1	0.48
	40-80	3962.47	7238.07	0.19	6630.97	14234.87	0.08
	80-120	2967.58	4866.17	0.03	5058.47	9299.47	0.08
	120-160	4674.5	6984.33	0.04	2077.43	3881.93	0.02

	160-200	1973.52	3867.29	0.02	1397.51	1743.57	0.01
	200-240	1049.7	2279	0.01	324.37	675.08	0.01
	Total	36347.47	60697.06	0.63	45374.18	76482.02	0.68
WT	0-40	18613.37	36364.1	0.35	27511.07	45699.57	0.48
	40-80	6805.04	7735.43	0.09	7787.8	15184.23	0.09
	80-120	2733	3557.1	0.03	2588.16	4755.53	0.03
	120-160	4020.95	4058.88	0.04	3145.02	5559.16	0.03
	160-200	3927.07	3698.25	0.04	1870	3596.03	0.02
	200-240	117.19	232.21	0.01	278.64	731.33	0.01
	Total	36216.62	55645.97	0.56	43180.69	75525.85	0.66

Table S7 Root distribution of TG (T349) and WT in different root layers during flowering stage

Lines	Soil distribution (cm)	Drought			Normal		
		root surface area (mm^2)	Root length (mm)	Root dry weight (g)	root surface area (mm^2)	Root length (mm)	Root dry weight (g)
TG	0-40	25274.87	44353.03	0.54	29940.25	59150.3	0.58
	40-80	6505	11718.4	0.11	7539.23	11998.73	0.12
	80-120	5533.06	8686	0.06	8833.47	10785.93	0.06
	120-160	4228.8	7435.33	0.06	5265.24	8627.33	0.04
	160-200	3240.22	6127.34	0.03	4637.35	7275.05	0.03
	200-240	2961.73	4036.01	0.04	2373.72	3894.91	0.02
	Total	47743.68	82356.11	0.84	58589.26	101732.25	0.85
WT	0-40	19315.47	36125.67	0.41	27442.17	42562.17	0.58
	40-80	3991.47	7566.7	0.05	7919.1	15183	0.05
	80-120	4356.2	9157.83	0.05	6623.49	12307.1	0.05

	120-160	3438.84	2366.5	0.02	4708.7	5155.57	0.04
	160-200	5217.83	7982.99	0.03	3968.65	4021.35	0.02
	200-240	2868.79	2905.65	0.01	4330.33	3836.4	0.02
	Total	39188.6	66105.34	0.57	54992.44	83065.59	0.76

Table S12 Soil water content during the growing season (2013)

Stages	Soil water content (%)													
	Shi jiazhuang						Ji nan				Lin fen			
	NIR		LIR		WIR		LIR		WIR		LIR		WIR	
	0-20 cm	20-40 cm	0-20 cm	20-40 cm	0-20 cm	20-40 cm	0-20 cm	20-40 cm	0-20 cm	20-40 cm	0-20 cm	20-40 cm	0-20 cm	20-40 cm
Over wintering	18.3±1.2	19.1±0.5	18.2±1.2	19.4±1.2	19.3±1.8	20.2±2.7	17.5±2.5	17.9±1.7	17.8±2.2	18.0±1.6	16.0±0.9	16.7±1.9	17.1±2.1	17.8±1.7
Turn green period	10.9±1.7	12.6±1.1	11.1±1.9	12.5±2.1	11.1±1.7	12.5±1.0	15.7±1.3	18.2±2.6	16.2±2.9	17.2±1.1	13.2±1.6	17.0±0.8	15.4±1.8	15.8±2.2
Jointing period	9.9±1.4	11.4±1.2	20.3±1.5	22.1±2.5	21.3±2.4	23.7±1.4	18.2±1.0	19.2±1.7	20.1±2.1	21.3±1.3	22.0±1.7	23.8±2.7	22.3±1.4	23.3±1.9
Heading period	7.8±1.2	10.4±1.8	18.9±1.2	20.4±3.1	18.0±1.7	19.4±1.2	16.4±2.6	19.5±1.7	18.6±2.4	19.9±1.4	14.9±2.2	17.1±2.1	17.8±1.4	19.0±1.7
Flowering period	7.5±1.2	9.5±1.7	15.2±1.9	16.4±2.2	22.1±1.7	24.6±2.3	14.8±2.4	16.0±1.9	20.1±1.9	21.3±1.7	13.7±2.7	15.3±1.6	20.4±1.9	23.0±2.6
Mature period	6.5±1.4	6.0±2.1	14.1±1.6	15.6±2.7	16.2±1.8	16.8±2.3	13.7±2.2	15.7±1.9	14.3±1.6	17.2±1.9	12.2±1.7	13.1±1.9	14.1±2.5	15.6±2.9

Table S13 Variety of plant height (PH) and panicle length (PL) under different irrigation conditions in the field.

Year	Lines	PH			PL		
		NIR	LIR	WIR	NIR	LIR	WIR
2010 - 2011	T349	69.8±1.5[a]	75.7±3.1[a]	81.0±1.3[a]	8.2±0.1[a]	8.3±0.1[a]	8.5±0.1[a]
	T398	69.0±0.4[a]	77.3±1.6[a]	81.0±1.6[a]	8.4±0.2[a]	8.4±0.1[a]	8.8±0.2[a]
	Jm19	68.8±1.5[a]	73.2±1.1[a]	83.2±2.3[a]	8.3±0.1[a]	8.3±0.1[a]	8.8±0.1[a]
2011 - 2012	CM3	68.3±0.6[a]	70.8±1.6[a]	75.4±1.6[a]	9.2±0.2[a]	9.2±0.1[a]	9.3±0.2[a]
	CM7	67.8±0.5[a]	72.0±0.3[a]	75.2±1.0[a]	8.9±0.1[a]	9.3±0.1[a]	9.2±0.2[a]

	**CM14**	**68.2±1.3 [a]**	**70.5±0.6 [a]**	**74.0±1.1 [a]**	**8.9±0.1 [a]**	**9.0±0.2 [a]**	**9.3±0.3 [a]**
	**Jm20**	**67.9±0.6 [a]**	**72.4±1.2 [a]**	**77.7±1.3 [a]**	**8.9±0.0 [a]**	**8.9±0.2 [a]**	**9.2±0.1 [a]**

PH, plant height; PL, panicle length. Data represent means ± SE of three replicates. Different letters indicate that the difference between the means of the transgenic lines and wild type was significant at the P < 0.05 (a, b and c) level.

Table S14 Main growth period of T4 generation transgenic wheat lines and WT under WIR in the 2012 growing season.

**Year**	**Lines**	**Shijiazhuang**					**Jinan**					**Linfen**				
		**TGP**	**JP**	**HP**	**FP**	**MP**	**TGP**	**JP**	**HP**	**FP**	**MP**	**TGP**	**JP**	**HP**	**FP**	**MP**
**2010 - 2011**	**T349**	**3/5**	**4/4**	**5/1**	**5/7**	**6/6**	**2/29**	**4/3**	**4/28**	**5/5**	**6/13**	**3/15**	**4/11**	**5/6**	**5/12**	**6/14**
	**T398**	**3/5**	**4/5**	**5/2**	**5/8**	**6/7**	**3/1**	**4/3**	**4/27**	**5/6**	**6/14**	**3/15**	**4/11**	**5/7**	**5/13**	**6/15**
	**Jm19**	**3/5**	**4/5**	**5/2**	**5/8**	**6/7**	**2/29**	**4/3**	**4/27**	**5/5**	**6/13**	**3/15**	**4/12**	**5/7**	**5/12**	**6/16**
**2011 - 2012**	**CM3**	**3/5**	**4/6**	**5/2**	**5/9**	**6/8**	**3/1**	**4/4**	**4/28**	**5/7**	**6/14**	**3/15**	**4/12**	**5/7**	**5/13**	**6/15**
	**CM7**	**3/5**	**4/6**	**5/2**	**5/9**	**6/8**	**3/1**	**4/4**	**4/28**	**5/7**	**6/14**	**3/15**	**4/12**	**5/7**	**5/13**	**6/15**
	**CM14**	**3/5**	**4/6**	**5/2**	**5/9**	**6/8**	**3/1**	**4/5**	**4/28**	**5/7**	**6/14**	**3/15**	**4/12**	**5/7**	**5/13**	**6/15**
	**Jm20**	**3/5**	**4/6**	**5/2**	**5/9**	**6/8**	**3/1**	**4/4**	**4/28**	**5/7**	**6/14**	**3/15**	**4/12**	**5/7**	**5/13**	**6/15**

TGP, Turn green period; JP, Jointing period; HP, Heading period; FP, Flowering period; MP, Mature period

Table S15 All primers for the stress-responsive genes

Primer name	Primer sequence (5' - 3')
PCR-UBI-F	AAAGAACGGGTAGAAGAAGTCCA
PCR-GmDREB1-R	GGCTTGAGATTGAGAGAGTTGGT
QRT-PCR-GmDREB1-F	CGATGAAACCTTACCGTGGAA
QRT-PCR-GmDREB1-R	AAGTCGGGCTTGAGATTGAG
COMT-F	CAAGGCAAGGTGGTAGTCGT
COMT-R	CCTGCGTAGATGTAGGTGGT
TDC-F	CAGTCGGCGTGTCCCATCTA
TDC-R	CATCGGCATCAGCTTGCATTA
SNAT-F	TCTATACTGCCATCATCATTCATCCA
SNAT-R	TCAACATTTCCGATCTGTCTCCA
WHAB1.6-F	GCAAGACTGCGGCAAAGG
WHAB1.6-R	CTCGGCGGCTCACCTAAT
GS1-F	ATGATCGCCGAGACCACCATCC
GS1-R	TCGTCCAAATCCTCCARTGGCC
GS2-F	GAACATGGACCCGTACACCGTG
GS2-R	TSAGGTCCTTCATACCTTCAGCG
βActin -F	CTCCCTCACAACAACCGC
βActin -R	TACCAGGAACTTCCATACCAAC
